# Restoration of WT1/miR-769-5p axis by HDAC1 inhibition promotes MMT reversal in mesenchymal-like mesothelial cells

**DOI:** 10.1038/s41419-022-05398-0

**Published:** 2022-11-17

**Authors:** Giulio Bontempi, Michela Terri, Sabrina Garbo, Claudia Montaldo, Davide Mariotti, Veronica Bordoni, Sergio Valente, Clemens Zwergel, Antonello Mai, Alessandra Marchetti, Alessandro Domenici, Paolo Menè, Cecilia Battistelli, Marco Tripodi, Raffaele Strippoli

**Affiliations:** 1 Department of Molecular Medicine, Sapienza University of Rome, Viale Regina Elena 324, 00161 Rome, Italy; 2 Department of Epidemiology, Preclinical Research and Advanced Diagnostics, National Institute for Infectious Diseases L. Spallanzani, IRCCS, Via Portuense, 292, Rome, 00149 Italy; 3 Department of Drug Chemistry and Technologies, Laboratory affiliated to Istituto Pasteur Italia-Fondazione Cenci Bolognetti, Sapienza University of Rome, Rome, Italy; 4 Renal Unit, Department of Clinical and Molecular Medicine, Sant’Andrea University Hospital, Sapienza University of Rome, 00189 Rome, Italy

**Keywords:** Mechanisms of disease, Chronic inflammation, miRNAs

## Abstract

Histone acetylation/deacetylation play an essential role in modifying chromatin structure and in regulating cell plasticity in eukaryotic cells. Therefore, histone deacetylase (HDAC) pharmacological inhibitors are promising tools in the therapy of fibrotic diseases and in cancer. Peritoneal fibrosis is a pathological process characterized by many cellular and molecular alterations, including the acquisition of invasive/pro-fibrotic abilities by mesothelial cells (MCs) through induction of mesothelial to mesenchymal transition (MMT). The aim of this study was to characterize the molecular mechanism of the antifibrotic role of HDAC1 inhibition. Specifically, treatment with MS-275, an HDAC1-3 inhibitor previously known to promote MMT reversal, induced the expression of several TGFBRI mRNA-targeting miRNAs. Among them, miR-769-5p ectopic expression was sufficient to promote MMT reversal and to limit MC migration and invasion, whereas miR-769-5p silencing further enhanced mesenchymal gene expression. These results were confirmed by HDAC1 genetic silencing. Interestingly, miR-769-5p silencing maintained mesenchymal features despite HDAC1 inhibition, thus indicating that it is necessary to drive MMT reversal induced by HDAC1 inhibition. Besides TGFBRI, miR-769-5p was demonstrated to target SMAD2/3 and PAI-1 expression directly. When analyzing molecular mechanisms underlying miR-769-5p expression, we found that the transcription factor Wilms’ tumor 1 (WT1), a master gene controlling MC development, binds to the miR-769-5p promoter favoring its expression. Interestingly, both WT1 expression and binding to miR-769-5p promoter were increased by HDAC1 inhibition and attenuated by TGFβ1 treatment. Finally, we explored the significance of these observations in the cell-to-cell communication: we evaluated the ability of miR-769-5p to be loaded into extracellular vesicles (EVs) and to promote MMT reversal in recipient mesenchymal-like MCs. Treatment of fibrotic MCs with EVs isolated from miR-769-5p over-expressing MCs promoted the down-regulation of specific mesenchymal targets and the reacquisition of an epithelial-like morphology. In conclusion, we highlighted an HDAC1-WT1-miR-769-5p axis potentially relevant for therapies aimed at counteracting organ fibrosis.

## Introduction

Mesothelial to mesenchymal transition (MMT) is a main cellular process implicated in the pathogenesis of chronic fibrotic pathologies of the serosal membranes of the body, such as peritoneal alterations during peritoneal dialysis (PD), chronic lung fibrosis, fibrotic adherences after abdominal surgery [1, 2]. Besides the functional alteration of the serosal membranes, peritoneal fibrosis offers the soil for the progression of tumors invading the peritoneal space, such as ovarian and colon carcinoma [3]. Thus, the study of molecular mechanisms controlling MC plasticity has both a basic and translational relevance.

Histone acetylation/deacetylation play an essential role in modifying chromatin structure and in regulating gene expression in eukaryotic cells, being key enzymes in this process, histone acetyltransferases (HATs) and histone deacetylases (HDACs). To date, eighteen distinct human HDACs have been reported; their impact on cell cycle regulation and on cellular plasticity made them attractive targets for drug discovery [4–6].

In the last years, many epigenetic inhibitors have been designed and are currently being validated, especially in the therapy of tumors and of nontumoral fibrotic pathologies [7]. Besides pan-HDAC inhibitors such as Trichostatin A and vorinostat, small molecules have been designed to selectively inhibit the activity of specific HDAC classes/isoforms [5].

Specifically, MS-275, an HDAC1-3 selective inhibitor, has recently been demonstrated to promote the reversal of fibrotic MCs from patients undergoing peritoneal dialysis (PD) towards an epithelial-like phenotype [8, 9]. Interestingly, treatment with MS-275 promoted TGFBRI downregulation in kidney and in mesothelium [9, 10].

In addition to chromatin epigenetic modifications, several noncoding RNAs, including micro-RNAs (miRNAs), have been demonstrated to control the expression of genes relevant for cell plasticity and fibrosis induction at a post-transcriptional level [11]. Moreover, miRNA expression itself may be epigenetically regulated by chromatin remodelers, adding further complexity to the regulation of cell plasticity.

The aim of this study was to characterize the molecular mechanism of the anti-fibrotic activity of HDAC1 inhibition, focusing on the regulation of miRNAs previously described to exert an anti-fibrotic activity. We elucidated the regulation and the functional role of a specific miRNA, miR-769-5p, here demonstrated to be sufficient to rescue an epithelial-like phenotype in PD patient-derived mesenchymal-like MCs. In particular, its ectopic expression was sufficient to induce MMT reversal and to limit migration and invasive activity in MCs. Mechanistically, miR-769-5p expression was demonstrated to be directly controlled by WT1, a master gene of MC differentiation that, in turn, is repressed by HDAC1 activity. Last, we demonstrated that miR-769-5p might be horizontally transferred to recipient cells via incorporation in extracellular vesicles (EVs), paving the way for further translational studies.

## Materials and methods

### Patients and cells

Effluent-derived MCs were isolated. From 10 clinically stable PD patients as described previously [12]. Baseline clinical data from these patients are reported in Table 1. Effluent-derived MCs were cultured in Earle’s M199 supplemented with 10% FBS (GIBCO® Life Technology, Monza, Italy), 2 mM L-glutamine (EuroClone), 100 U/ml penicillin, 100 µg/ml streptomycin (Gibco-Life Technologies) and amphotericin B (2,5 µg/ml). The purity of Effluent-derived MCs was determined as in ref. [13].

**Table 1:** List of PD patients enrolled in this study.

Patients	Sex	Age	Cause of kidney failure	Diabetes	Hypertension	Months on PD	PD technique	Exchanges	PD solution	glucose (mg/dl)	d/p creat 4 h PET	Peritonitis	Hemoperitoneum	Escapes	Epitheliod/non-epitheliod
1	M	56	malignant hypertension	no	yes	40	CAPD	1	FMC stay-safe balance®	2270	0,48	no	no	no	E
2	M	69	unknown	no	yes	44	CAPD	4	FMC stay-safe balance®	1580	0,45	no	no	no	E
3	F	85	p-ANCA vasculitis	no	yes	58	CAPD	3	FMC stay-safe balance®	1815	0,5	no	no	no	NE
4	M	75	Ig A GNF	no	yes	92	APD	15 liters/night	FMC stay-safe balance®	1360	0,67	no	no	no	NE
5	M	61	diabetes/ hypertension	yes	yes	12	CAPD	1	FMC stay-safe balance®	2270	0,63	no	no	yes	E
6	F	66	p-ANCA vasculitis	no	yes	31	CAPD/CCPD	4/20 liters/24 h	FMC stay-safe balance®	2270	0,66	no	no	no	E
7	M	66	Chronic pielonephritis	no	yes	72	CAPD	2	FMC stay-safe balance®	2270	0,66	2	no	no	NE
8	M	53	ADPKD	no	yes	6	CAPD	3	FMC stay-safe balance®	2270	0,5	2	no	yes	E
9	M	54	unknown	no	yes	13	CAPD	1	FMC stay-safe balance®	1360	0,71	no	no	no	E
10	M	64	Ig A GNF	no	yes	55	CCPD	25 liters/24 h	FMC stay-safe balance®	1580	0,68	2	no	yes	NE
11	M	57	type 1 diabetes mellitus	yes	yes	56	CCPD	20 liters/24 h	FMC stay-safe balance®	1815	0,54	no	no	no	E

The human mesothelial cell line MeT‐5 A (ATCC, Rockville, MD) was cultured in Earle’s M199 as above (except for amphotericin B); Cells were grown at 37 °C in a humidified atmosphere with 5% CO_2_. In some experiments to enhance MMT-like features, effluent-derived MCs and Met-5A were treated with TGFβ1 (2 ng/ml). The cytokine dose used is in the range of those detected in peritoneal-dialysis fluids from patients with peritonitis [14] and is similar to those used in previous studies [15, 16]).

Experiments on effluent-derived MCs were performed according to guidelines from the ethics committee of Sant’Andrea Hospital, Sapienza University (Rome, Italy). Written informed consent was obtained from all PD patients. The protocol and informed consent were reviewed and approved by the Ethics Committee of Clinic Investigation of Sapienza University ref: 4697_2017 (Roma, Italy).

### Antibodies and chemicals

Mouse monoclonal antibody (mAb) anti-TGFβ1 1D11.16.8 (BE0057) was from inVivoMab/Bio X Cell (Lebanon, NH); mAbs anti-HDAC (10E2), -SNAIL (L70G2), -ALIX (3A9) were from Cell Signalling (Danvers MA); mAbs anti-PAI-1 (sc-5297), -CD9 (sc-59140), -TUBULIN (sc-32293), -HSP90 (sc-13119), -WT1 (sc-7385), -CALNEXIN (sc-23954) -GAPDH (sc-32233) were from Santa Cruz Biotechnology (Dallas, TX); mAb anti-α−SMA (A52228) was from SIGMA ALDRICH (Saint Louis, MO); -ANNEXIN-VII (610668) was from BD-Transduction Laboratories (Franklin Lakes, NJ); rabbit mAb anti-SYNTHENIN (AB133267) was from ABCAM (Cambridge, UK).

Rabbit polyclonal antibody (pAb) anti-TGF-β Receptor Antibody type I (ABF17-I) was from Merck Life Science S.r.l (Darmstadt, Germany); pAb anti-WT1 (12609-I-AP), was from Proteintech (Chicago, IL); pAbs anti-SMAD2/SMAD3 (31025), -phospho-SMAD2/3 (8828) were from Cell Signalling; pAb anti-FIBRONECTIN (AB2413) was from ABCAM. MS-275, used at the concentration of 250 nM, was from the Mai laboratory.

### Western Blotting

Monolayers of effluent-derived MCs or MeT-5A cells were lysed in CelLytic (C2978, SIGMA ALDRICH), were quantified by Bradford protein Assay (5000001 from Biorad Hercules, CA) boiled for 5’ at 95 °C and were loaded on 10% acrylamide gels. Gels were electrophoresed at 100 V in Running Buffer (25 mM Tris, 190 mM glycine; 0.1% SDS) and then transferred to a nitrocellulose membrane (Pure Nitrocellulose Membrane 0.45 μm; Bio-Rad) at 15 V for 50’ in Transfer Buffer (Tris-Glycine buffer 10% from Biorad, 20% Methanol). Blots were blocked in 5% non-fat milk prepared in PBS-0.05% Tween and incubated overnight with the primary antibody. The day after, the blots were incubated with HRP- conjugated species-specific secondary antibodies (Goat Anti-Mouse IgG (H + L) HRP Conjugated (170-6516) or Goat Anti-Rabbit IgG (H + L)-HRP Conjugated (172-1019, Bio-Rad). Nitrocellulose-bound antibodies were detected by chemiluminescence with ECL, and the signal was revealed through ChemiDoc Imaging system (Biorad). Full-length western blots are shown as ‘Supplemental material’.

### Immunoprecipitation

For immunoprecipitation, cells were lysed (50 mM Tris-HCl at pH8, 150 mM NaCl, 1% NP-40, 0.5% Sodium deoxycholate, 0.1% SDS), added with 1 mM PMSF, PIC 1:200 (P-8340 from Sigma), 10 mM NaF, 1 mM NaV, 200 mM NaMo for 30 min. Cell lysates were centrifuged for 15 min at 13000 rpm at 4 C. Supernatants were mixed with the specific antibody (1 μg per sample) or control IgG for 2 h, and protein G–agarose beads were added for a further 2 h. Beads were washed with lysis buffer and processed for western blotting.

### Reverse-transcriptase polymerase chain reaction

mRNAs and miRNAs extracted from cell cultures with miRNeasy Mini Kit (QIAGEN Cat. 217004). MiRNAs were reverse transcribed with MystiCQ microRNA cDNA Synthesis Kit (MIRRT-100-RXN from SIGMA ALDRICH) according to the manufacturer’s instructions. mRNAs were reverse transcribed with Takara Prime Script RT Mastermix (RR036A-1) from Takara (Kusatsu, Japan).

cDNAs were amplified by qPCR reaction using GoTaq® qPCR Master Mix (Promega, Madison, WI, USA), and the reactions were carried out in BioRad-iQ-iCycler. The results were analyzed with CFX Manager software (Biorad), and the relative amounts obtained with 2^(−ΔCt)^ method were normalized with respect to the gene L34 and miR-16. The specific primer pairs are listed in Table 2.

**Table 2:** List of PCR primers used in this study.

Name	Sequences
SMAD2	F 5′-ACAGCTAGGCAGGGCAACTA-3′ R 5′-GGGCAGAGTTCACAGTCACA-3′
SMAD3	F 5′-CCCCAGAGCAATATTCCAGA-3′ R 5′-GACATCGGATTCGGGGATAG-3′
HDAC1	F 5′-CATCGCTGTGAATTGGGCTG-3′ R 5′-CCCTCTGGTGATACTTTAGCAGT-3′
COL-1A1	F 5′-AGCCAGCAGATCGAGAACAT-3′ R 5′-TCTTGTCCTTGGGGTTCTTG-3′
OCCLUDIN	F 5′-AAGGTCAAAGAGAACAGAGCAAGA-3′ R 5′-TATTCCCTGATCCAGTCCTCCTC-3′
WT1	F 5′-CACAGCACAGGGTACGAGAG-3′ R 5′-CAAGAGTCGGGGCTACTCCA-3′
ACTA2	F 5′-CAGCCAAGCACTGTCAGG-3′ R 5′-CCAGAGCCATTGTCACAC-3′
PAI-1	F 5’-AGTGGACTTTTCAGAGGTGGA-3’ R 5’-GCCGTTGAAGTAGAGGGCATT-3’
TGFβ1	F 5’-AAGGACCTCGGCTGGAAGGTG-3’ R 5’-CCCGGGTTATGCTGGTTGTA-3’
TGFβRI	F 5′-AACTTCCAACTACTGGCCCT-3′ R 5′-GGTGAATGACAGTGCGGTTG-3′
L34	F 5’-GTCCCGAACCCCTGGTAATAG-3’ R 5’-GGCCCTGCTGACATGTTTCTT-3’
miR-16-5p	5’-TAGCAGCACGTAAATATTGGCG-3’
miR 98-5p	5’-TGAGGTAGTAAGTTGTATTGTT-3’
miR-490-3p	5’-CAACCTGGAGGACTCCATGCTG-3’
miR 769-5p	5’-TGAGACCTCTGGGTTCTGA-3’
miR 3607-3p	5’-ACTGTAAACGCTTTCTGATG-3’
miR 5195-3p	5′-ATCCAGTTCTCTGAGGGGGCT-3′
U6	F 5’-CTCGCTTCGGCAGCACA-3’ R 5’-AACGCTTCACGAATTTGCGT-3’

### Luciferase assay

The sequences of wild type and mutant type of SMAD 2, SMAD3, and PAI-1 3’UTR were cloned into pmirGLO Dual-Luciferase miRNA Target Expression Vector (Promega, Madison, WI, USA). MeT5A were seeded in 12-well plates and co-transfected with the reporter vectors and miR-769-5p mimic or miR-NC. Dual-Luciferase Reporter Assay System (Promega) was used to analyze the luciferase activity.

### Chromatin immunoprecipitation assay (ChIP)

ChIP analysis was performed as previously reported [17]. 5 μg of anti-WT1 or rabbit IgG were used. After washes, samples were eluted with the elution buffer (NaHCO_3_ 100 mM, SDS 1%), treated with 10 µg of RNase A and with 240 µg of proteinase K (Sigma-Aldrich). The extracted DNA was used in the qPCR analyses. The following specific primer pairs were used: for miR-769-5p promoter WT1 binding sites 1-2 5′ACAGCTGCCTCTGTGGTCTC3′ 5′AGTGCGTCCCCTCCCTAC3′ and for miR-769-5p promoter WT1 binding site 3 5′CTGTGTTTCTGCGTGCTTTC3′ and 5′CTGGGCGAGACCAGGAGA3′. Data were expressed as (IP-IgG)/Input. The analysis of WT1 binding sites on the miR-769 promoter was performed by PROMO software using version 8.3 of TRANSFAC. The analysis of acetylation peaks on miR-769 promoter was provided by ENCODE using UCSC Genome Browser on Human (GRCh37/hg19).

### siRNA-mediated knockdown and ectopic expression

100 × 10^3^ MCs were seeded on 12-well plates 24 h prior to transfection.

Cells were transfected with either 30 pmol of ON-TARGET plus siRNAs SMART POOL (Cat. L-003493–00) against human HDAC1, *mir*Vana® miRNA inhibitor of miR-769-5p (5′AGCUCAGAACCCAGAGGUCUCA3′) (Cat. 4464084, Assay ID MH19974) or the same amount of ON-TARGETplus Non-targeting siRNA #1 (Cat. D-001810-01-50) and 2 μl Lipofectamine® RNAiMAX Reagent from Thermo Fisher Scientific (Waltham, MA USA) in 200 μl Optimem from Gibco (Waltham, MA USA). 1 ml of supplemented medium per well was also added. The transfection lasted 48 h. Knockdown efficiency was determined by RT-PCR and western blot.

For miR-769-5p ectopic expression, the same amount of MCs was transfected with 30 pmol of *mir*Vana® miRNA mimic (5′UGAGACCUCUGGGUUCUGAGCU3′) (Cat. 4464066, Assay ID MC19974) and the same amount of miRVana miRNA mimic Negative Control #1 (Cat#4464058).

### Confocal microscopy and immunofluorescence

MCs were washed in cold PBS, fixed with 4% paraformaldehyde (Sigma-Aldrich) in PBS, and permeabilized with 0.2% Triton X-100 (Sigma-Aldrich) in PBS. Alexa Fluor 488 secondary antibody was from Thermo Fisher Scientific; Cy3-conjugated secondary antibody was from Jackson Immunoresearch (Philadelphia, PA). Draq5 was used to visualize nuclei (Invitrogen). Coverslips were mounted in Prolong Gold antifade (Life Technologies) and examined under a confocal microscope (Leica TCS SP2, Wetzlar, Germany). Digital images were acquired with the Leica software. A minimum of 4 fields per sample (at least 100 total cells per total) from three independent experiments was analyzed.

### Scratch assay

MCs were allowed to reach 100% confluency. Serum-reduced cells (0.5% FBS) were left untreated or pre-treated with TGF-β1 (2 ng/ml); after 24 h, cells were treated with mimic negative control or ectopically expressed with miR-769-5p (30 nm) for 48 h. Alternatively, cells were treated with MS-275 (250 nM) or with DMSO. After 24 h, cells were treated with siRNA negative control or transfected with miR-769-p5 specific siRNA for 24 h. Then, a scratch wound was created using a p200 tip [18]. For microscopy time-lapse experiments, micrographs were taken every 30 min from time 0 to 18 h after the beginning of the scratch (only the 6 h time is shown). Tree-independent experiments were performed. Images were acquired using a Leica THUNDER 3D Live Cell Imaging System (Leica Application Software (LAS) X 3.7.2; Leica Microsystems) using THUNDER Computational Clearing Settings at 5X magnification. Cell-devoid areas at time 0 and 6 hours after the scratch were quantified through the Fiji Image J image processing package.

### Invasion assay

For transwell invasion assays, 8-μm pore. 6.5 mm Insert 24-well cell-culture plates (Corning Inc) coated with type I collagen (0.1 mg/mL; Upstate Biotechnology) were used. 25×10^3^ cells were plated in the upper chamber in serum-reduced medium (1% FBS); in the lower chamber, M199 medium was supplemented with 20% FBS as a chemoattractant. Cells were fixed with 100% MetOH, stained with Crystal violet solution, and counted by CellProfiler software. At least 1.000 cells were counted in control samples. MCs were allowed to invade for 6 hours. Four independent experiments from MCs derived from four different PD patients were performed.

### EVs isolation and validation

The isolation of cell-derived EVs from exo-depleted conditioned culture medium was performed by ultracentrifugation according to guidelines from the International Society for Extracellular Vesicles [19]. Briefly, 15 ml of conditioned medium collected from a p150 dish were centrifuged at 2.000 g for 20 min at 4 °C to remove cells, at 20.000 g for 30 min at 4 °C to remove macrovesicles. The medium was filtered (0.22 μm) and then ultracentrifuged at 100.000 g for 70 min at 4 °C. The pellet was resuspended in PBS. Size and concentration of the obtained vesicles were measured by IZON (Accela, Praha, Czech Republic). The expression of EVs markers and negative controls (CD9, FLOTILLIN1, ALIX, SYNTHENIN, ANNEXIN-VII, and CALNEXIN) was analyzed by WB.

### Statistical analysis

Statistical significance was determined with a t‐test (one tailed) using GraphPad Prism version 8.0 (La Jolla, CA, USA). Differences were considered significant *: *P* < 0.05; ** *P* < 0.01; *** *P* < 0.001.

## Results

### HDAC1 inhibition promotes the expression of anti-fibrotic miRNAs targeting TGFBRI mRNA

**Fig. 1:**
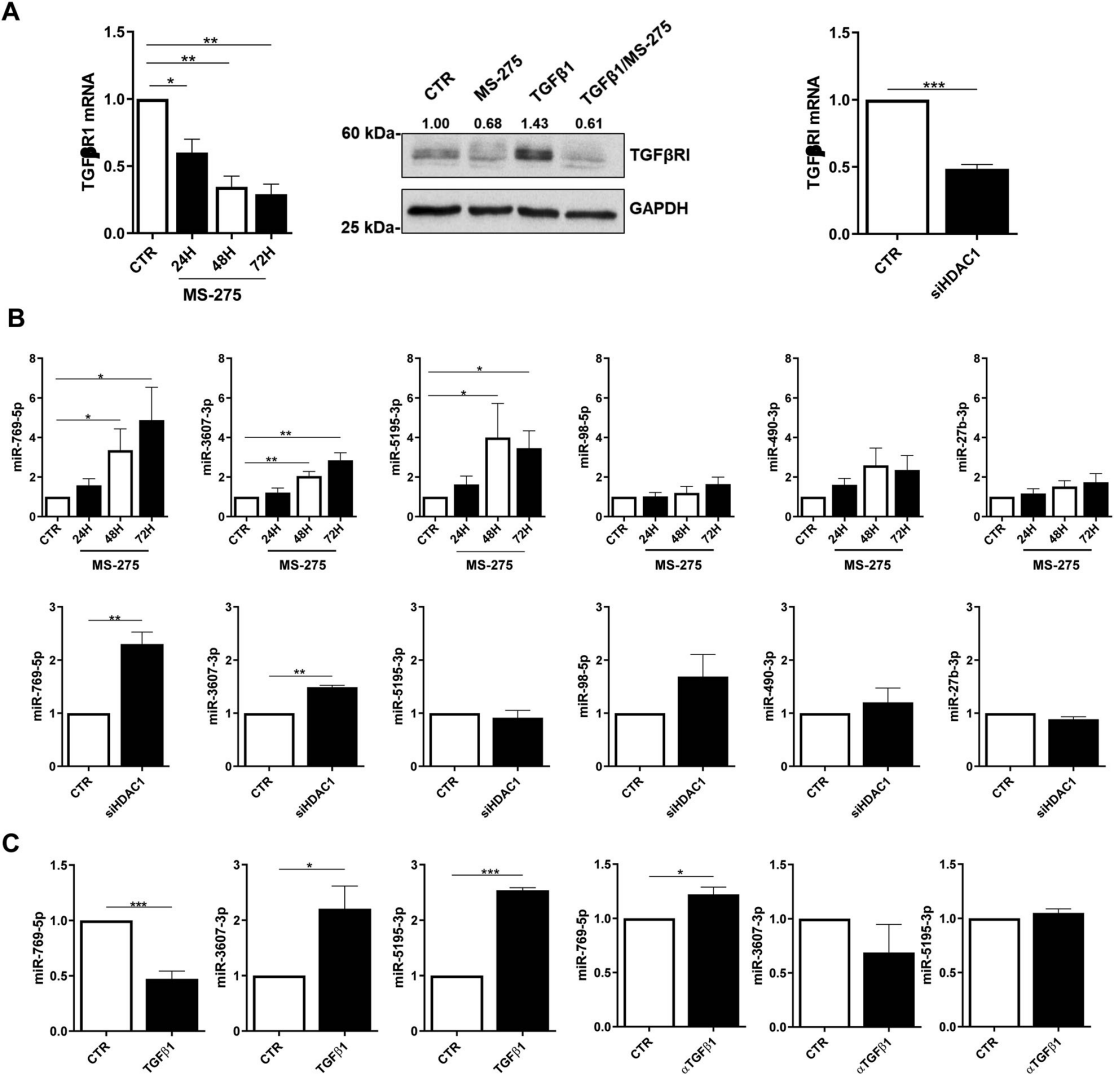
HDAC1 inhibition promotes the expression of antifibrotic miRNAs targeting TGFBRI mRNA. **A left**, RT-PCR showing the expression of TGFBRI mRNA in MCs from PD patients treated with MS-275 (250 nM for, 24, 48 and 72 h). Quantitative RT-PCR was performed on total RNA. L34 mRNA levels were used for normalization. Bars represent the mean ± SEM of triplicate determinations in four independent experiments. **middle**, WB showing the expression of TGFBRI from the same experiment. GAPDH was used as a loading control. Data are representative of three independent experiments. **right**, RT-PCR showing TGFBRI mRNA expression upon HDAC1 genetic silencing using a specific siRNA. L34 mRNA levels were used for normalization. Bars represent the mean ± SEM of duplicate determinations in four independent experiments. **B top**, RT-PCR showing the expression of miR-769-5p, miR3607-3p, miR5195-3p, miR-98-5p, miR-490-3p and miR-27b-3p upon exposure to MS-275 at the same conditions as in **A** miR-16 was used for normalization. Bars represent the mean ± SEM of duplicate determinations in five independent experiments. **Bottom**, the expression of the same miRNAs was analyzed upon HDAC1 genetic silencing. miR-16 was used for normalization. Bars represent the mean ± SEM of duplicate determinations in four independent experiments. **C** RT-PCR showing the expression of miR-769-5p, miR3607-3p and miR5195-3p upon treatment with TGFβ1 (2 ng/ml for 72 h) (**top**), or upon treatment with anti-TGF-β1 monoclonal antibody 1D11.16 (100 μg/ml). (**bottom**). Bars represent the mean ± SEM of duplicate determinations in three to five independent experiments.

HDAC1 inhibition by MS-275 has been demonstrated to promote the reacquisition of epithelial-like features in mesenchymal MCs from PD patients having undergone MMT in vivo (Rossi et al., 2018). As shown in Fig. 1, in the same experimental setting, the pharmacological inhibition of HDAC1 promotes TGFBRI mRNA and protein downregulation (Fig. 1A**, left, middle**). The same result has been obtained by HDAC1 genetic silencing (Fig. 1A**, right and** Suppl. Figure 1).

In order to shed light on the mechanisms promoting MMT reversal in a context of HDAC1 inhibition, the role of anti-fibrotic miRNAs was hypothesized, and the expression of 6 miRNAs (miR-769-5p, miR3607-3p, miR5195-3p miR-98-5p, miR-490-3p, and miR-27-3p) candidates to target TGFBRI by TargetScan cross-search with the existing literature was analyzed. Among them, miR-769-5p, miR3607-3p, and miR5195-3p were significantly induced by MS-275 (Fig. 1B**left**), whereas only miR-769-5p and miR3607-3p were significantly induced by genetic silencing of HDAC1(Fig. 1B**right**) in mesenchymal-like MC from PD patients.

Next, the effect of TGFβ1 on the expression of these miRNAs was analyzed. Among the three analyzed miRNAs, only miR-769-5p expression was significantly downregulated by treatment with TGFβ1 and rescued by TGFβ1 immune depletion **(**Fig. 1C).

Overall, these observations pinpoint miR-769-5p, which is at the same time repressed by TGFβ1 and inhibitor of TGFBRI, as a relevant regulator of this pathway.

### miR-769-5p genetic silencing reverses the effect of HDAC1 inhibition on mRNA expression of TGFBRI and other MMT-related markers

**Fig. 2:**
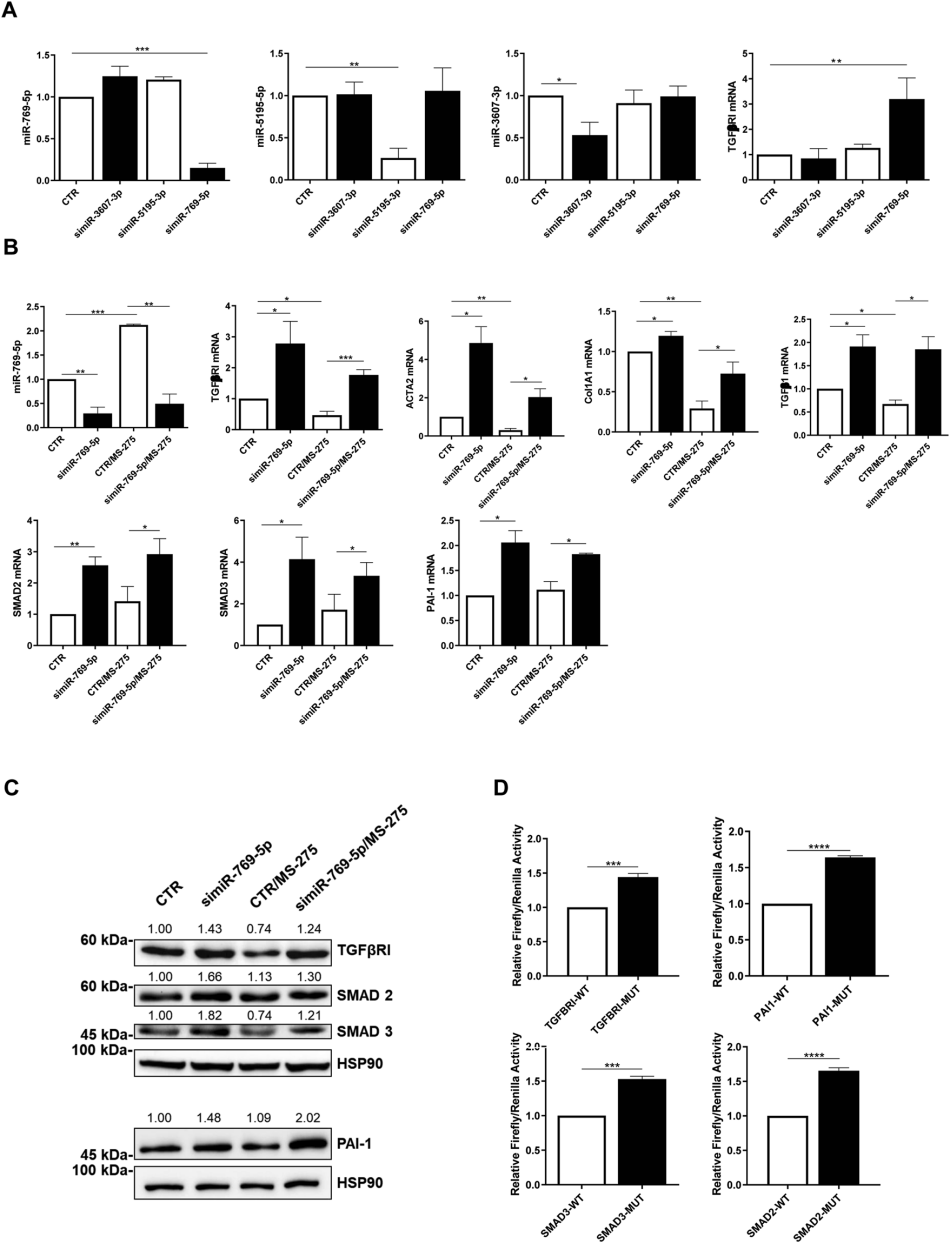
miR-769-5p genetic silencing reverses the effects of MS-275 in the expression of TGFβRI and other MMT-related markers. **A** RT-PCR showing the effect of miR-769-5p, miR3607-3p and miR5195-3p specific silencing on TGFBRI mRNA expression in MCs from PD patients. miR-16 was used for normalization. Bars represent the mean ± SEM of duplicate determinations in three to five independent experiments. **B** RT-PCR showing the effect of miR-769-5p genetic silencing on the expression of TGFBRI, ACTA2, COL1A1, TGFβ1, SMAD2, SMAD3 and PAI-1 in the presence of MS-275. Quantitative RT-PCR was performed on total RNA. L34 mRNA levels were used for normalization. Bars represent the mean ± SEM of duplicate determinations in three to four independent experiments. **C** WB showing the expression of TGFBRI, PAI-I, SMAD2 andSMAD3 upon genetic silencing of miR-769-5p. HSP90 was used as a loading control. Data are representative of three independent experiments. **D** Dual luciferase reporter gene assay showing the specific targeting of TGFBRI, SMAD2, SMAD3 and PAI-1, by miR-769-5p Bars represent the mean ± SEM of duplicate determinations in four to five independent experiments performed in MeT5A cells.

The functional effects of miR-769-5p, miR3607-3p, and miR5195-3p on TGFBRI mRNA were then analyzed by genetic silencing. Among these three miRNAs, in line with the results shown in Fig. 1, only miR-769-5p silencing effectively enhanced TGFBRI expression in mesenchymal-like MC from PD patients (Fig. 2A).

To provide a broader observation, the effect of miR-769-5p on the expression of MMT markers was analyzed by both genetic silencing and by ectopic expression as a mirror experiment.

miR-769-5p silencing was found to promote a further expression of mesenchymal markers. Interestingly, the downregulation of TGFBRI, ACTA2, COL1A1, and TGFβ1, induced by treatment with MS-275, was reversed by miR-769-5p genetic silencing. The expression of other markers (SMAD2, SMAD3, PAI1) was upregulated by miR-769-5p silencing independently on MS-275 activity (Fig. 2B). The upregulation of mesenchymal markers observed after miR-769-5p genetic silencing was also confirmed at protein level by WB (Fig. 2C)

Since complementary miR-769-5p target sequences were found in SMAD2, SMAD3, and PAI 3’UTR mRNA, luciferase experiments were performed to demonstrate direct interaction. Indeed, miR-769-5p was demonstrated to bind directly to the 3’UTR of SMAD2, SMAD3, and PAI1 mRNA. A direct binding to TGFBRI mRNA was also confirmed (Fig. 2D).

### miR-769-5p ectopic expression is sufficient to inhibit TGFβ1-induced MC migration and invasion

**Fig. 3:**
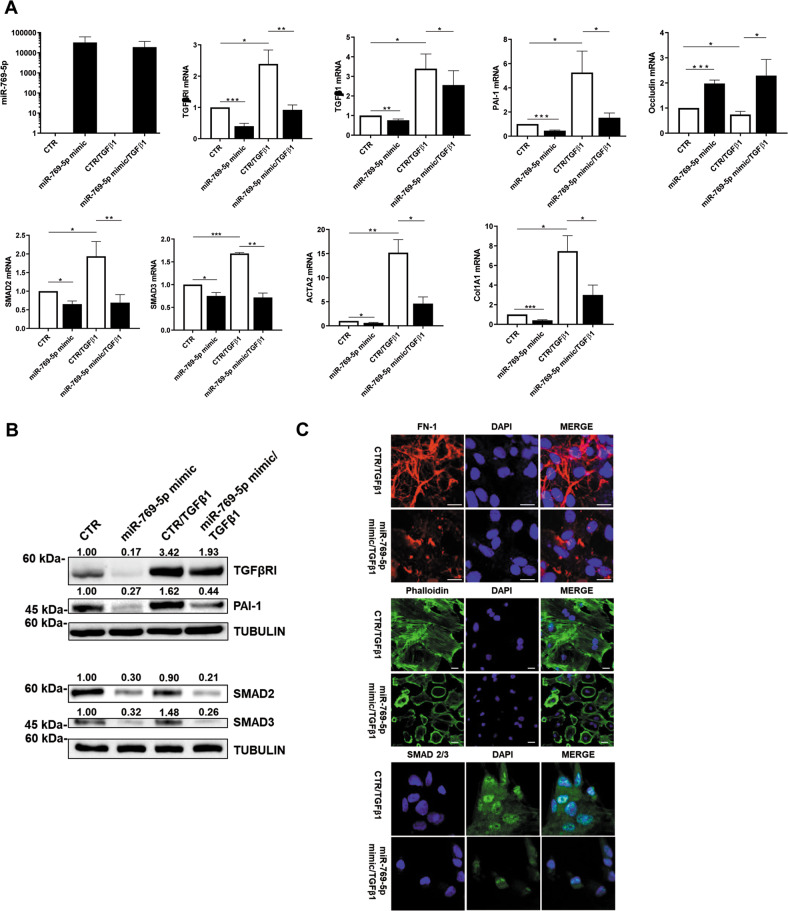
miR-769-5p ectopic expression is sufficient to inhibit TGFβ1-induced expression of MMT molecular markers. **A** RT-PCR experiments showing the expression of miR-769-5p and of TGFBRI, TGFβ1, PAI-1, Occludin-1, SMAD2, SMAD3, ACTA2, and Col1A1 mRNA upon ectopic expression of miR-769-5p. Quantitative RT-PCR was performed on total RNA. L34 mRNA levels were used for normalization. Bars represent the mean ± SEM of duplicate determinations in three to six independent experiments. **B** WB showing the expression of SMAD2, TGFBRI, SMAD3 and PAI-I upon ectopic expression of miR-769-5p. Tubulin was used as a loading control. Data are representative of three independent experiments. **C** Immunofluoresce of MCs from PD patients transfected with mimic miR-769-5p. Fixed cells were stained with antibodies against type I COLLAGEN, FN-1 and α-SMA. Data are representative of three independent experiments.

**Fig. 4:**
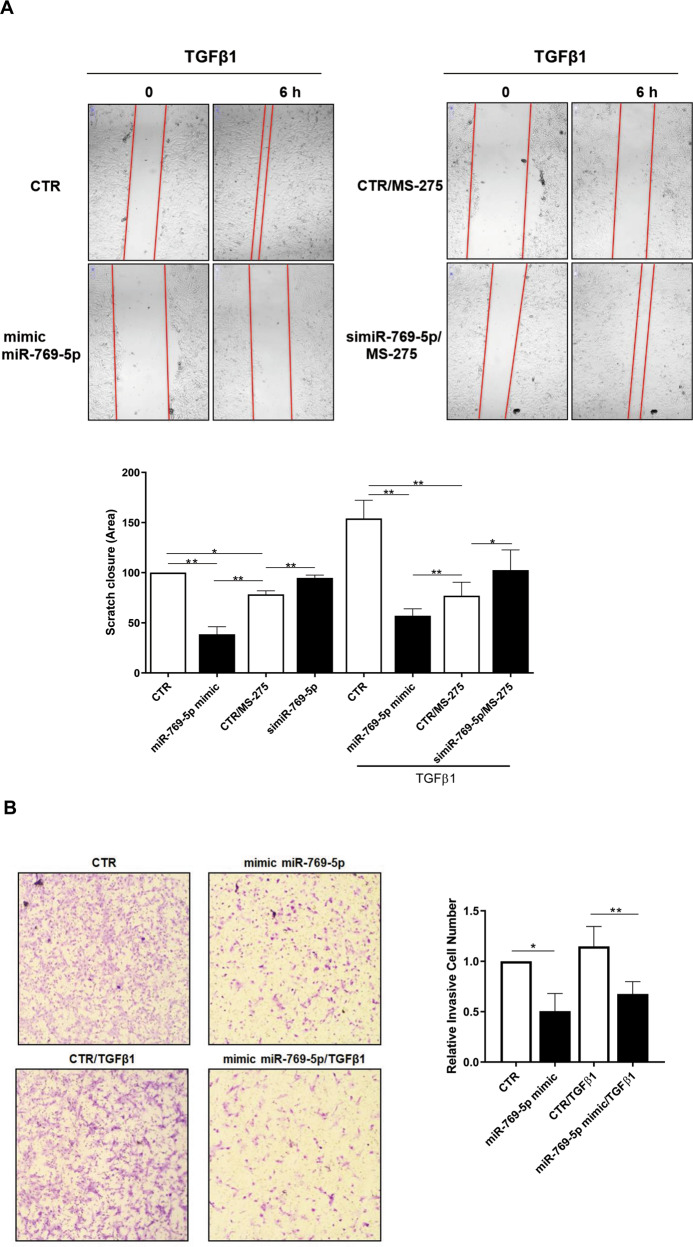
miR-769-5p ectopic expression is sufficient to inhibit TGFβ1 induced MCs migration and invasion. **A left**, Effect of miR-769-5p genetic silencing and ectopic expression in the presence of MS-275 on wound closure. MCs from patients undergoing PD were allowed to reach 100% confluency. MCs were pre-treated with DMSO, MS-275 for 48 h in culture medium supplemented with 10% FCS. Cells were then transfected with si-miR-769-5p or with mimic miR-769-5p in the presence or absence of TGFβ1 and a scratch was performed with a 200 μl tip. Therefore, microphotographs were taken at time 0 and 18 h after the scratch. Representative experiment is shown of TGFβ1 treated MCs of three performed. **Right**, Histogram showing a quantification of the experiment described. **B left**, Effect of miR-769-5p ectopic expression in the presence of TGFβ1 on invasion through type I COLLAGEN matrices. Representative experiment is shown one of three performed experiments. **Right**, histogram resuming the results of the experiment described.

In line with these results, miR-769-5p ectopic expression, irrespectively of concomitant TGFβ1 stimulation, was found to promote the downregulation of mesenchymal markers as well as OCCLUDIN epithelial marker upregulation (Fig. 3A). The downregulation of mesenchymal markers after the miR-769-5p ectopic expression was also confirmed at protein level by WB (Fig. 3B). Confocal microscopy experiments demonstrated both the reacquisition of an epithelial-like morphology and the downregulation of mesenchymal markers COLLAGEN I, FN-1 and α−SMA (Fig. 3C). Overall, these observations pinpoint miR-769-5p, as pivotal in the HDAC1-mediated mesenchymal marker regulation. To demonstrate a functional role of miR-769-5p in MC migration and invasion, miR-769-5p was either genetically silenced or ectopically expressed in MCs and analyzed in a scratch assay (Fig. 4A**top and** Suppl. Figure 1). A quantification of these experiments is shown in Fig. 4A**bottom**.

In this experimental condition, ectopic expression of miR-769-5p promoted a delay in scratch closure. Interestingly, the effect of HDAC1 inhibition on scratch closure was proven to be dependent on miR-769-5p since it was reverted by miR-769-5p silencing. Notably, the miR-769-5p effect on migration was also reproduced in the presence of TGFβ1 (Fig. 4A).

The analysis of the role of miR-769-5p on cell migration was extended to cellular invasion. Representative images of MC migration and invasion are shown in Fig. 4B**, left**. In this experimental condition, miR-769-5p ectopic expression limited MCs invasion on type I COLLAGEN matrix, both in basal conditions and upon TGFβ1 treatment (Fig.4B).

Overall, these results demonstrate that HDAC1 controls *(i)* the expression of MMT molecular markers, (*ii)* migration, and *(iii)* invasion of fibrotic primary MCs in dependence on the pivotal role of miR-769-5p.

### WT1-mediated induction of miR769-5p is repressed in fibrotic MCs by HDAC1 activity

**Fig. 5:**
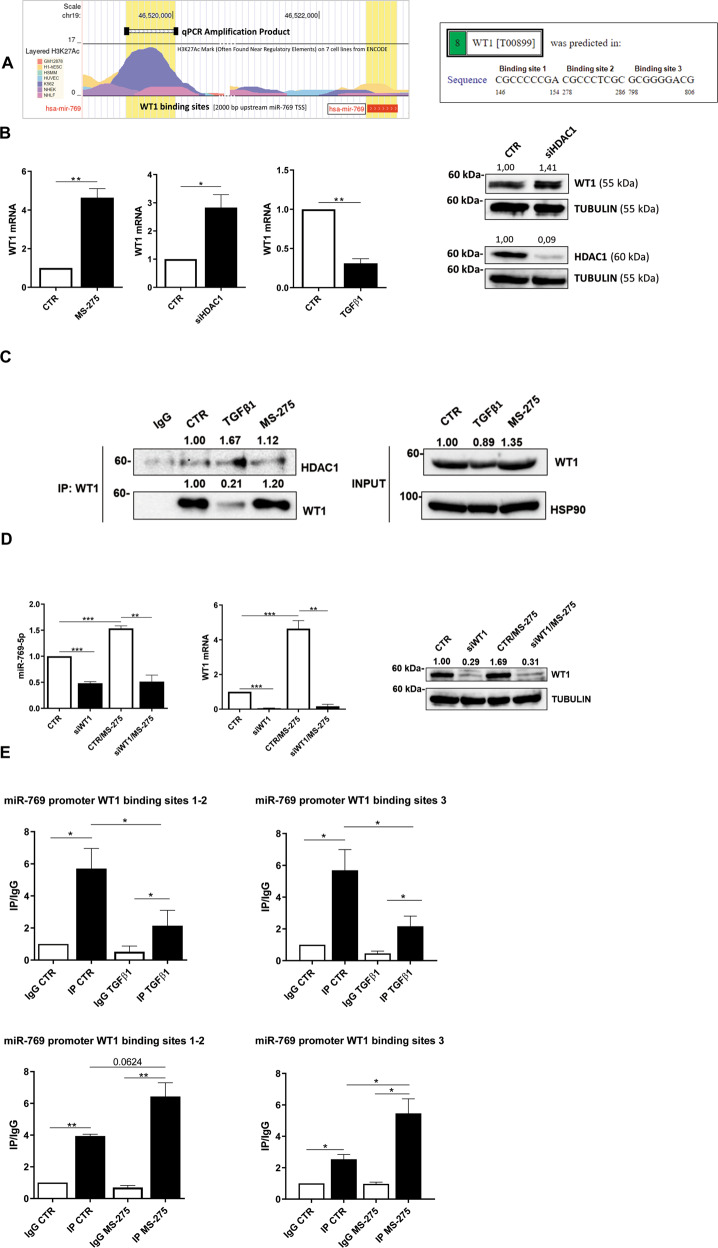
WT1-mediated induction of miR769-5p is repressed in fibrotic MCs by HDAC1 activity. **A left**, predicted WT1 binding site on miR-769-5p promoter by PROMO. **Right**, study of H3K27 acetylation of miR-769-5p promoter from ENCODE. **B Left**, RT-PCR showing WT1 mRNA expression upon stimulation with TGFβ1, after HDAC1 genetic silencing, and treatment with MS275 (250 nM for 72 h). Bars represent the mean ± SEM of duplicate determinations in three to four independent experiments. **Right**, WB showing expression of WT1 (top) and HDAC1 (bottom) upon HDAC1 genetic silencing. Tubulin is used as a loading control. Data are representative of three independent experiments. **C left**, immunoprecipitation of WT1 showing HDAC1-WT1 interactions upon stimulatin with TGFβ1 and treatment with MS275. A WB of input cell lysates showing WT1 expression and HSP90 as a loading control is shown in the right of the figure. Data are representative of three independent experiments. **D** RT-PCR showing the expression of miR-769-5p (**left**) and of WT1 (**middle**) upon miR-769-5p genetic silencing and treatment with MS-275. Bars represent the mean ± SEM of duplicate determinations in four independent experiments. **Right**, WB showing the expression of WT1in the same conditions as in **D left**. Tubulin is used as a loading control. Data are representative of three independent experiments. **E** qPCR of ChIP assays with anti-WT1 and as control, with normal rabbit IgG on chromatin from MCs from PD patients treated with treated with TGFβ1 for 24 h (**top**) or with MS-275 for 72 h (**bottom**) or left untreated (NT) and when indicated. Data show increased binding of WT1 to specific binding sites (1-3) at miR769-5p promoter. Bars represent the mean ± SEM of duplicate determinations in three independent experiments.

We, therefore, focused on the molecular mechanism controlling miR-769-5p expression by HDAC1 inhibition. Bioinformatic analysis and previously gathered experimental evidence suggest a role for WT1, a transcription factor promoting MCs differentiation. Indeed, the analysis of the miR-769-5p promoter by PROMO revealed the presence of three potential binding sites (Fig. 5A**left**). Moreover, the study of functional elements of the miR-769-5p promoter from ENCODE revealed the presence of multiple acetylation peaks overlapping with the predicted WT1 binding sites in 7 cell lines analyzed (Fig. 5A**right**). Interestingly, WT1 expression inversely correlated with both HDAC1 pharmacological inhibition and genetic silencing, while it was inhibited by treatment with TGFβ1 (Fig. 5B**left**). WT1 protein induction upon HDAC1 genetic silencing was also confirmed by WB analysis (Fig. 5B**right**). Moreover, coimmunoprecipitation experiments demonstrated that WT1 and HDAC1 colocalize in basal conditions and upon treatment with TGFβ1 (Fig. 5C). These results suggest a direct effect of HDAC1 in repressing WT1 expression and transcriptional activity. These results prompted us to asses a possible direct causal role of WT1 activity on miR-769-5p expression in a context of HDAC1 inhibition. As shown in Fig. 5D, WT1 silencing abolished miR-769-5p induction upon treatment with MS-275. Finally, chromatin immunoprecipitation (ChIP) experiments performed on the three predicted consensus sites demonstrated a direct binding of WT1 to the miR-769-5p promoter (Fig. 5E**left, middle**). Interestingly, TGFβ1 (previously shown downregulating miR-769-5p expression, Fig. 1C) also reduced WT1/miR-769-5p promoter interactions, thus further indicating a positive activity of WT1 on miR-769-5p expression. As expected, treatment with MS-275 increased WT1 binding to the miR-769-5p promoter **(**Fig. 5E**right**). Overall, these experiments indicate that HDAC1 in fibrotic MCs represses miR-769-5p expression impairing WT1 binding on its promoter.

### EV transfer of miR-769-5p promotes MMT reversal

**Fig. 6:**
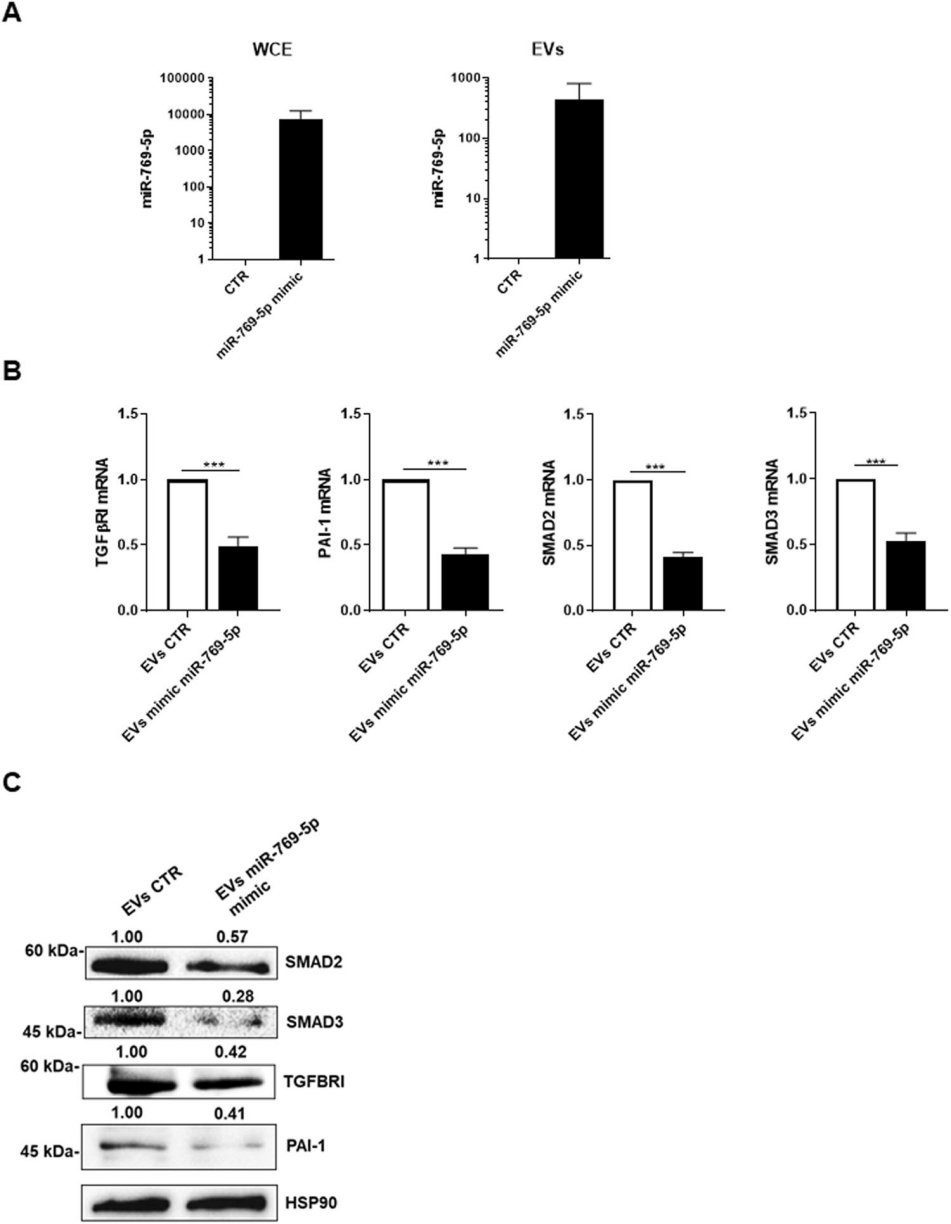
EV transfer of miR-769-5p promotes MMT reversal. **A** RT-PCR showing expression of miR769-5p in whole cell extract (WCE) (**left**) or the EV fraction (**right**) upon miR769-5p ectopic expression in primary MCs from PD patients. miR-16 was used for normalization. Bars represent the mean ± SEM of duplicate determinations in three independent experiments **B** RT-PCR showing expression of TGFβ1, PAI-1, SMAD2 and SMAD3 mRNA from total RNA of recipient MCs after treatment with EVs (10 μg) from MCs transfected with control or mimic miR769-5p. Bars represent the mean ± SEM of duplicate determinations in six independent experiments. **C** WB showing the expression of Smad2, Smad3, TGFBRI and PAI-1 in MCs from PD patients after treatment with EVs expressing miR769-5p (generated as Fig. 6A). Hsp90 was used as a loading control. Data are representative of three independent experiments.

Sequence analysis of miR-769-5p revealed the presence of cis elements described to impact miRNA compartmentalization. Specifically, we found three EXO-motifs, small sequences associated with EV export of miRNAs, while no CL/CELL motifs associated with cytoplasmic retention were observed (Suppl. Figure2) [20–22]. Moreover, data from the literature reported that miR-769-5p is loaded into osteosarcoma-derived EVs [23]. In this frame, we wondered whether the here demonstrated reprogramming activity of miR-769-5p could be horizontally transferred via EVs. EVs isolated by primary MCs expressing miR-769-5p were structurally characterized (Suppl. Figures 3, 4A/B) [19]. miR-769-5p was confirmed in EVs obtained by MCs and markedly increased upon miR-769-5p ectopic expression in MCs (Fig. 6A).

Functionally, the incubation of primary mesenchymal-like MCs with EVs derived from producing MCs ectopically expressing miR-769-5p resulted in a significant reduction of mesenchymal markers TGFBRI, PAI-1, SMAD2, and SMAD3 mRNA and protein expression (Fig. 6B, C). These results demonstrated that the anti-fibrotic activity of miR769-5p may be horizontally transferred by EV delivery.

Overall, these results highlight that: (i) miR-769-5p is relevant in counteracting MCs mesenchymal-like state, (ii) a HDAC1/WT1 molecular axis regulates the miR-769-5p expression, and (iii) miR-769-5p ectopic expression promotes MMT reversal beyond cell-autonomous mechanisms.

## Discussion

The aim of this study was to characterize molecular mechanisms of anti-fibrotic activity exerted by HDAC1 inhibition. Focusing on the anti-fibrotic miR-769-5p, we elucidated its role in MMT reversal. Moreover, we dissected a molecular mechanism underlying its expression, identifying a previously unrecognized HDAC1-WT1-miR-769-5p axis. Last, we found that the activity of miR-769-5p may be horizontally transferred to fibrotic MCs, opening translational implications. A quality of the present study is the fact that it has been performed on primary cells from PD patients. Indeed, the limited availability of cell cultures with respect to standard cell lines and the increased variations of biological values between samples from different patients are compensated by the quality of ex vivo studies compared with the greater concordance of results obtained from primary cells with pathogenic mechanisms effectively occurring during fibrosis induction in PD in patients.

Several miRNAs have been described so far to impact peritoneal fibrosis [24, 25]. In particular, some miRNAs were characterized for their inhibitory effect on fibrosis: miR-15 was described to suppress inflammation and fibrosis of MCs through VEGF inhibition [26]; miR-30a negatively regulated TGF-β1-induced EMT and peritoneal fibrosis by targeting Snai1 [27]; miR-9-5p suppressed pro-fibrogenic transformation of fibroblasts and MCs from PD patients [28]; miR-302c modulated peritoneal dialysis-associated fibrosis by targeting CTGF [29]; miR-29b inhibited peritoneal fibrosis in a mouse model of PD [30].

On the other hand, other miRNAs were demonstrated to promote peritoneal fibrosis: miR-199a/214 Cluster targeting E-cadherin and Claudin-2 promoted high glucose-induced peritoneal fibrosis [31]; miR-145 promoted MMT during the development of peritoneal fibrosis by suppressing FGF10 activity [32], miR-21 promoted fibrogenesis during PD [33].

Specifically, miR-769-5p has previously been linked to inhibition of invasive activity and to reduced survival in tumors [34]. miR-769-5p suppressed cell proliferation, migration, and invasion targeting TGFBRI mRNA in non-small cell lung carcinoma [35]. Conversely, in osteosarcoma, it has been demonstrated to promote cell proliferation, invasion and metastasis by targeting the phosphatase DUSP16 [23].

To our knowledge, miR-769-5p has never been associated with events related to MCs plasticity and peritoneal fibrosis. Our study demonstrates that miR-769-5p ectopic expression is sufficient to inhibit the expression of several MMT-related genes, MCs migration, and invasion. This observation, mechanistically linked to the direct targeting of TGFBRI, SMAD2/3, and PAI-1 expression, opens to translational implications.

Our study provides a link between miR-769-5p expression and HDAC1 activity. This parallels previous observations where HDAC inhibition was found to modulate the expression of other miRNAs. Vorinostat and panobinostat, class I and II HDAC inhibitors, increased miR-146a expression, promoting an anti-inflammatory activity [36]. Pan HDAC inhibitors promoted the expression of several miRNAs, affecting metastasis formation [37]. Genome-wide analysis revealed selective modulation of microRNAs and mRNAs by valproic acid, a class I and IIa HDAC inhibitor, impacting B lymphocyte differentiation. [38]. In another study, trichostatin A, a pan-HDAC inhibitor, altered microRNA expression profiles in apoptosis-resistant breast cancer cells [39].

MS-275 has already been demonstrated to exert biological functions through the induction of miRNAs. MS-275 upregulated miR-34a, inhibiting pulmonary arterial remodeling [40]. Through the expression of miR-205, the same inhibitor was demonstrated to improve the effect of chemotherapeutics in cancer cells [41]. Moreover, it was demonstrated to promote the downregulation of erbB2/erbB3 through the expression of miR-125a, miR-125b, and miR-205. Interestingly, probably due to cellular/tissue specificity, miR-769-5p was not intercepted so far in genome-wide analyses performed upon HDAC pharmacological inhibition.

MS-275 is an inhibitor of HDAC1 and, to a lesser extent, of HDAC2 and HDAC3 activity. At the concentration used in this study (250 nM), this pharmacologic inhibitor is specific for the HDAC1 isoform [42]. Indeed, the effect of MS-275 on MMT reversal was reproduced by HDAC1 genetic silencing.

In this frame, we attributed a pivotal role to the zinc finger transcriptional factor WT1 in the regulation of miR-769-5p expression. WT1 is expressed in all stages of kidney development, while its expression becomes restricted to podocytes in the mature kidney. In the same cells, WT1 expression is downregulated by TGFβ1 through hyper-methylation of the WT1 promoter [43, 44]. WT1 is also a main transcription factor regulating MC development and differentiation and is expressed in mature peritoneum and pleura [45, 46]. Its expression may be helpful in tracking mesothelial cells having migrated to the sub-mesothelial stroma from the pleura/peritoneum monolayer upon inflammatory/pro-fibrotic conditions [47]. The role of WT1 in MMT dynamics is somehow controversial: besides being considered a positive regulator of MC differentiation and a promoter of the epithelial-like state [45], it has been reported as a driver of myofibroblast activation in pleura fibrosis [48]. In embryonic mesothelial-derived liver cells, WT1 depletion enhanced both fibrogenesis after injury and induction of myofibroblastic transition [49]. This activity is highly evocative of what is reported in hepatocytes for HNF4a: its depletion has been found to cause mesenchymal gene expression both in vitro and in animal models. As for HNF4a in other contexts, therefore, WT1 may be considered as a master regulator of epithelial cell identity in mesothelium [50]. With respect to miRNAs, WT1 was demonstrated to promote the expression of an array of miRNAs and to cooperate in post-transcriptional inhibition of the epigenetic regulator EZH2 in mesenchymal stem cells [51]. Our results link WT1 activity to the induction of an anti-fibrotic program through the induction of miR-769-5p expression.

Thus, we highlight the existence of a homeostatic axis involving WT1-miR-769-5p expression promoting the maintenance and rescue of an epithelial-like identity in MCs. This axis appears to be attenuated in conditions of high TGFβ1/active HDAC1, which has been found in many fibrotic experimental settings. Interestingly, ectopic expression of miR-769-5p may contribute to the re-establishment of an epithelial-like phenotype in the fibrotic peritoneum.

Notably, this last observation has been extended to the possibility of horizontally transferring miR-769-5p via EVs. miRNA compartmentalization in EVs is a tightly regulated process dependent on RNA binding proteins (RBPs) recognition of miRNAs short sequences targeting cytoplasm retention or EV delivery [20–22]. Interestingly, miR769-5p express three EXO motifs bona fide favoring its EV export. We found that ectopic miR769-5p expression resulted in an efficient miR769-5p loading in EVs, allowing the downregulation of mesenchymal-related genes in recipient primary fibrotic MCs.

**Fig. 7:**
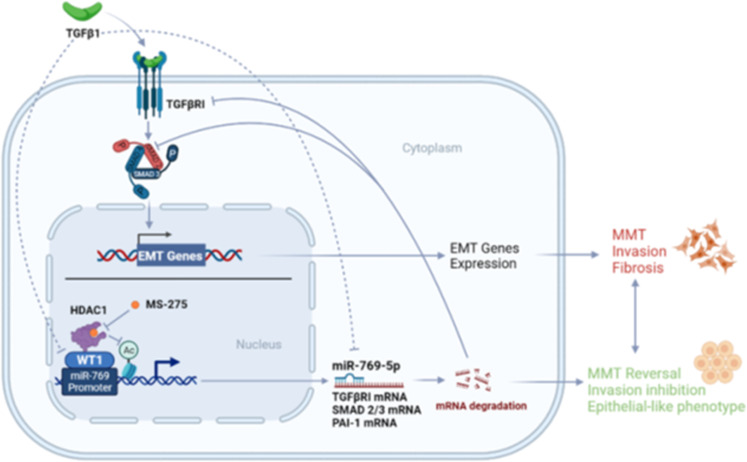
Both TGFβ1 and HDAC1 limit WT1 activity in mesenchymal-like MCs. In this condition, the antifibrotic miR-769-5p is downregulated. HDAC1 inhibition may promote MMT reversal through WT1-induced miR-769-5p expression.

Overall, this study identifies the previously unreported HDAC1-WT1-miR-769-5p axis controlling epithelial-mesenchymal dynamics in MCs (Fig. 7). This molecular axis (blocked by HDAC1 inhibition) favors the repression of WT1 activity in conditions of high TGFβ-1, a signature virtually found in all the known cases of peritoneum fibrosis.

Growing capacity to exploit RNA-based approaches shall benefit from basic science reports exploring the ability of specific anti-fibrotic miRNAs to reestablish an epithelial identity in MCs to counteract peritoneal fibrosis.

## Supplementary information

supplemental figures

Supplementary figure legends

Extended Data (western blot raw data)

aj-checklist

## Data Availability

All datasets generated and analysed during this study are included in this published article and its Supplementary Information files. Additional data are available from the corresponding author on reasonable request.
