# Supplementary material for: Restoration of WT1/miR-769-5p axis by HDAC1 inhibition promotes MMT reversal in mesenchymal-like mesothelial cells
Source: Cell Death Dis. 2022 Nov 17;13(11):965. doi: 10.1038/s41419-022-05398-0 (PMC9672101; doi:10.1038/s41419-022-05398-0)
Supplement: Supplementary file 1 — supplemental figures [file 41419_2022_5398_MOESM1_ESM.pptx]

## Slide 1
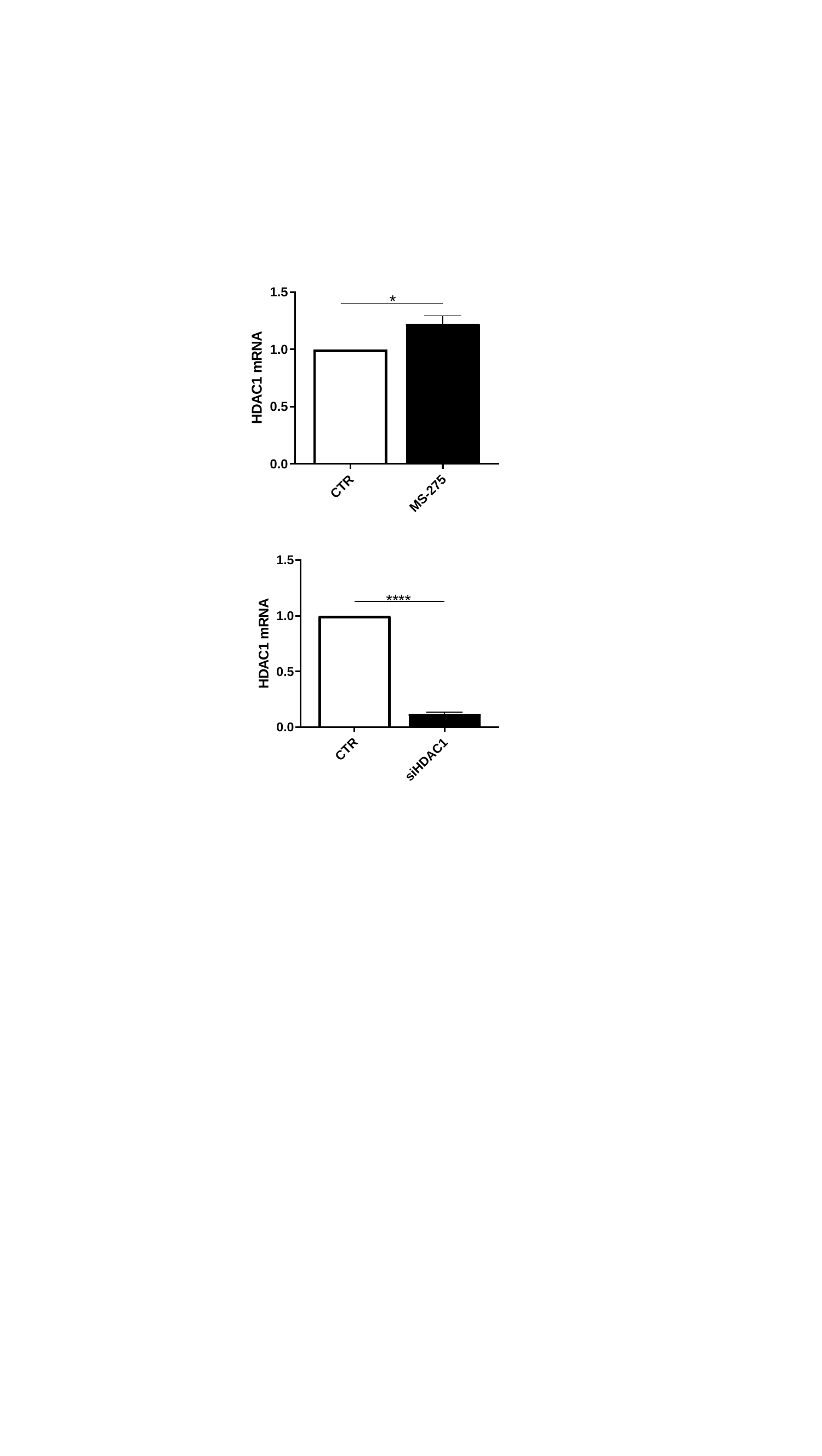

## Slide 2
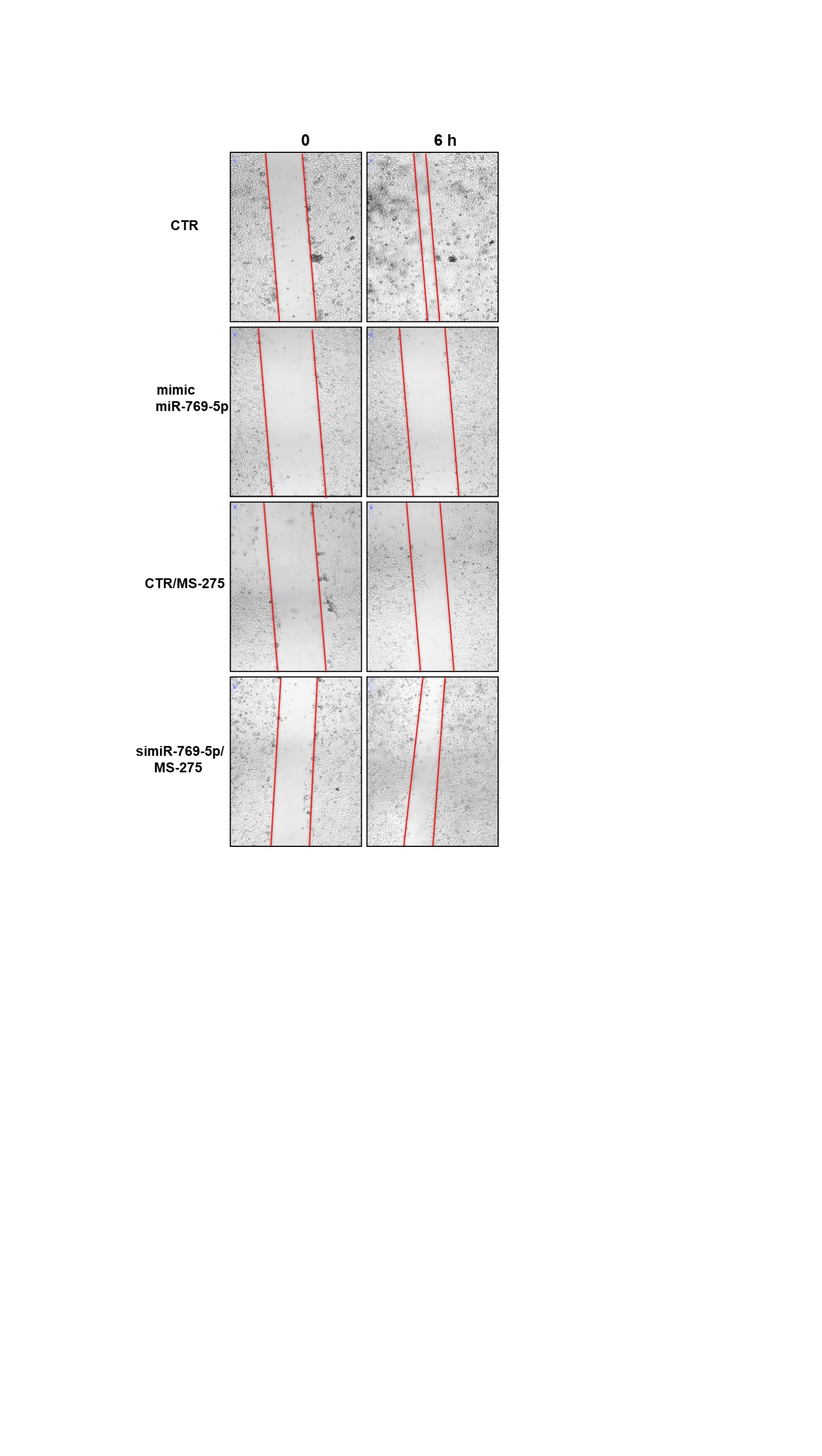

## Slide 3
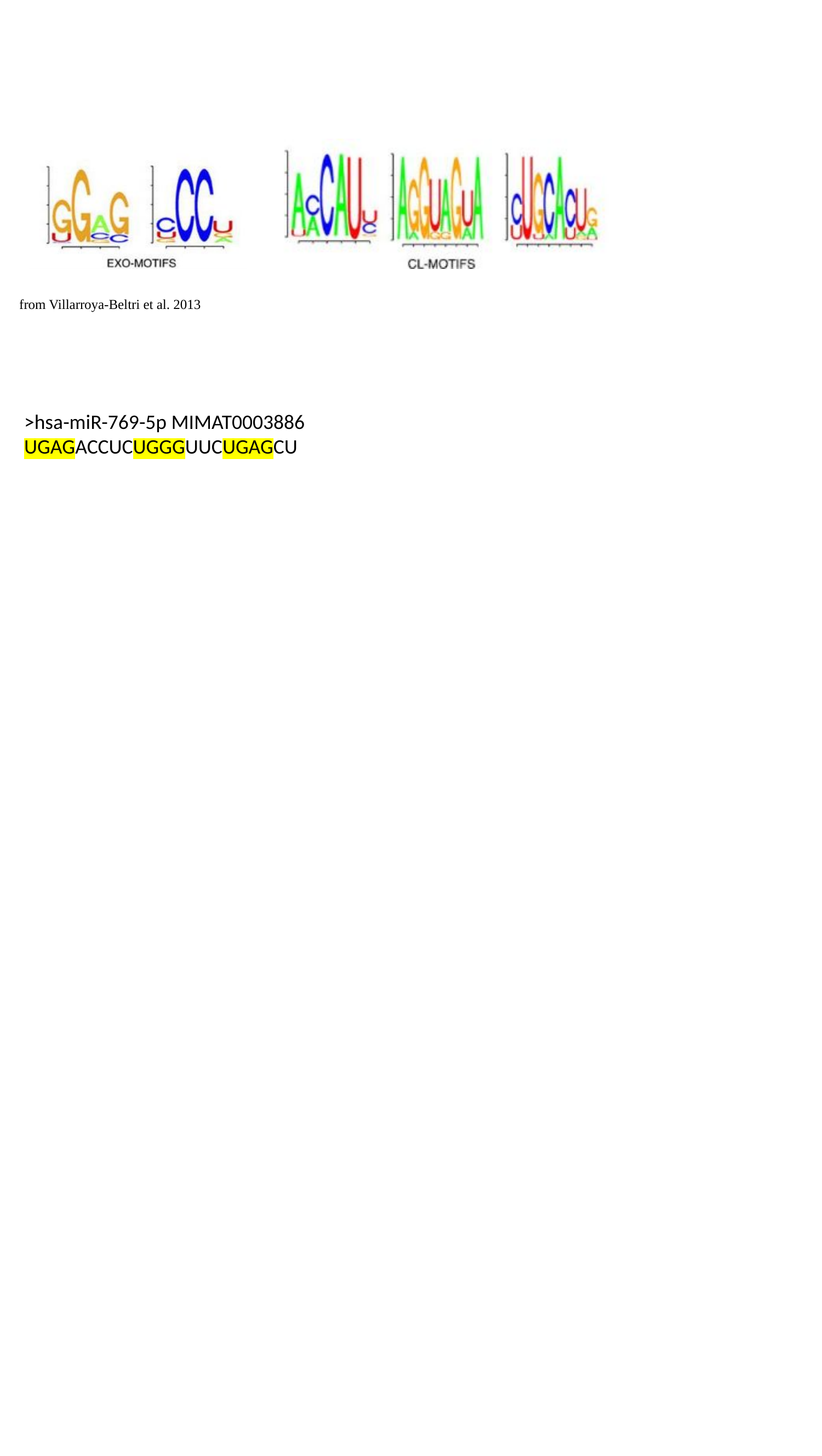

from Villarroya-Beltri et al. 2013
>hsa-miR-769-5p MIMAT0003886
UGAGACCUCUGGGUUCUGAGCU

## Slide 4
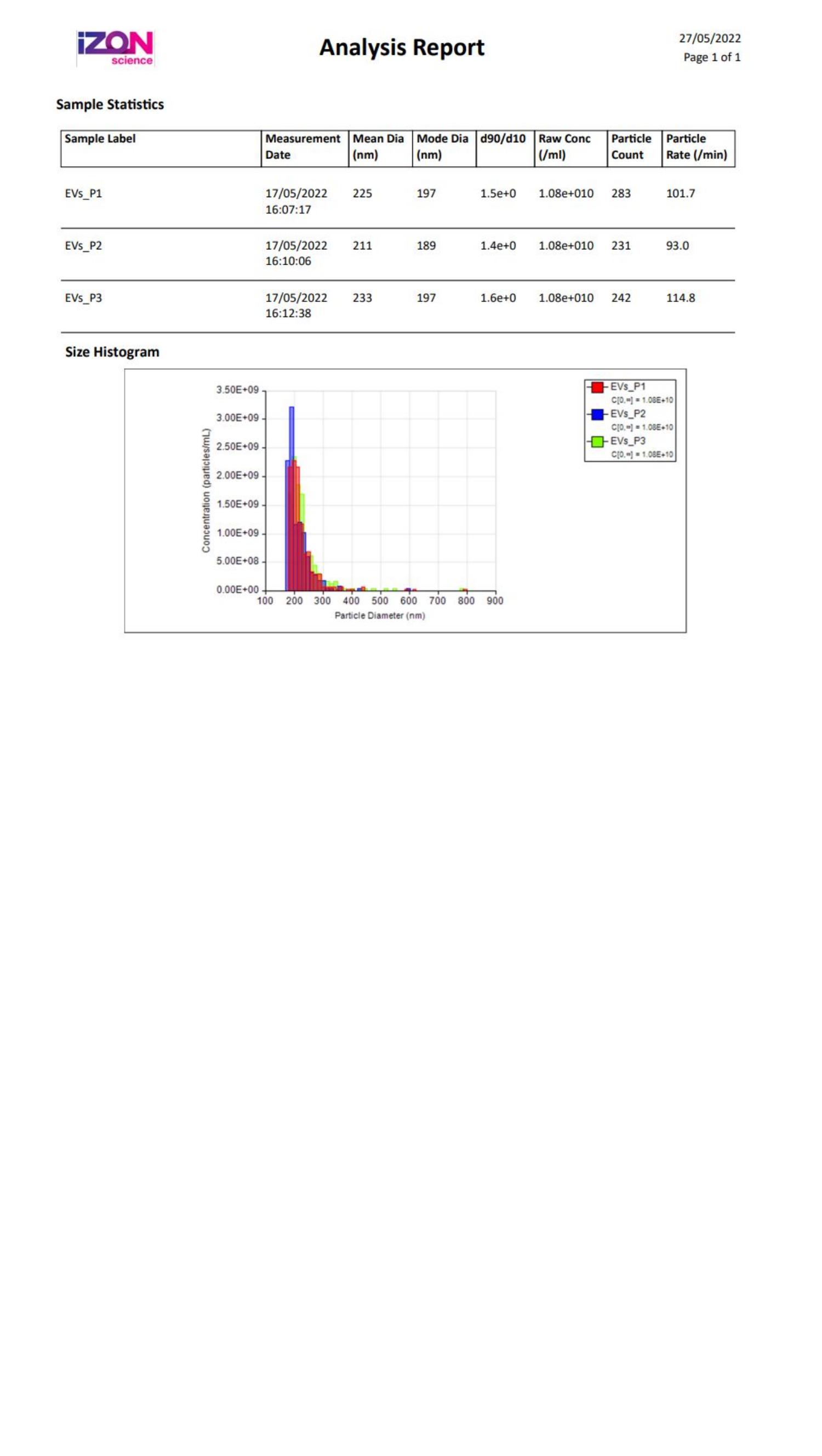

## Slide 5
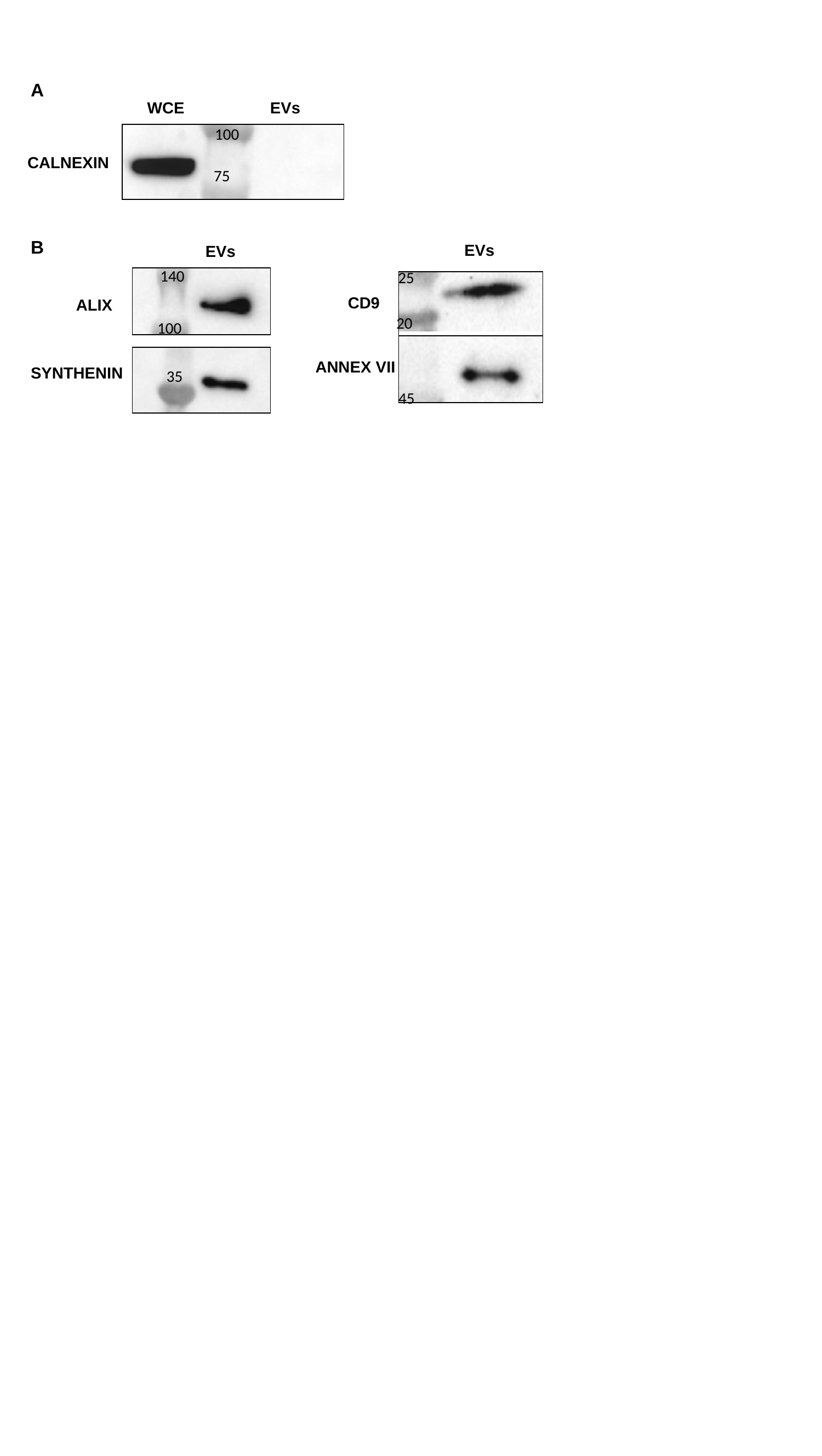

A
WCE
EVs
100
CALNEXIN
75
B
EVs
EVs
140
25
CD9
ALIX
20
100
ANNEX VII
SYNTHENIN
35
45
