# Supplementary material for: Restoration of WT1/miR-769-5p axis by HDAC1 inhibition promotes MMT reversal in mesenchymal-like mesothelial cells
Source: Cell Death Dis. 2022 Nov 17;13(11):965. doi: 10.1038/s41419-022-05398-0 (PMC9672101; doi:10.1038/s41419-022-05398-0)
Supplement: Supplementary file 2 — Supplementary figure legends [file 41419_2022_5398_MOESM2_ESM.docx]

**Supplementary figure legends**

**Supplementary figure 1.** qRT-PCR showing the expression of HDAC1 in MCs from PD patients treated with MS-275 (250 nM) for 48 h or genetically silenced for HDAC1. Quantitative RT-PCR was performed on total RNA. L34 mRNA levels were used for normalization. Bars represent the mean ± SEM of duplicate determinations in three independent experiments.

**Supplementary figure 2.** Representative images of the experiment described in Fig. 4A Representative experiment of three performed showing CTR treated MCs (without TGFβ1 treatment).

**Supplementary Figure 3.** Top, cartoon showing nucleotide sequences of EXO and CL motifs

Bottom: the three EXO motifs within miR-769-5p sequence are shown.

**Supplementary figure 4.** Representative MCs derived EV characterization (size and concentration) by IZON science device.

**Supplementary figure 5.** Biochemical characterization of EVs from MCs from PD patients. **A** WB showing the expression of CALNEXIN from whole cell lysates (WCE) and EVs from MCs from PD patients. **B** WB showing the expression of ALIX, SYNTHENIN, CD9 and ANNEX VII from EVs released by MCs from PD patients. Data are representative of three independent experiments.
