# Supplementary material for: Restoration of WT1/miR-769-5p axis by HDAC1 inhibition promotes MMT reversal in mesenchymal-like mesothelial cells
Source: Cell Death Dis. 2022 Nov 17;13(11):965. doi: 10.1038/s41419-022-05398-0 (PMC9672101; doi:10.1038/s41419-022-05398-0)
Supplement: Supplementary file 3 — Extended Data (western blot raw data) [file 41419_2022_5398_MOESM3_ESM.pptx]

## Slide 1
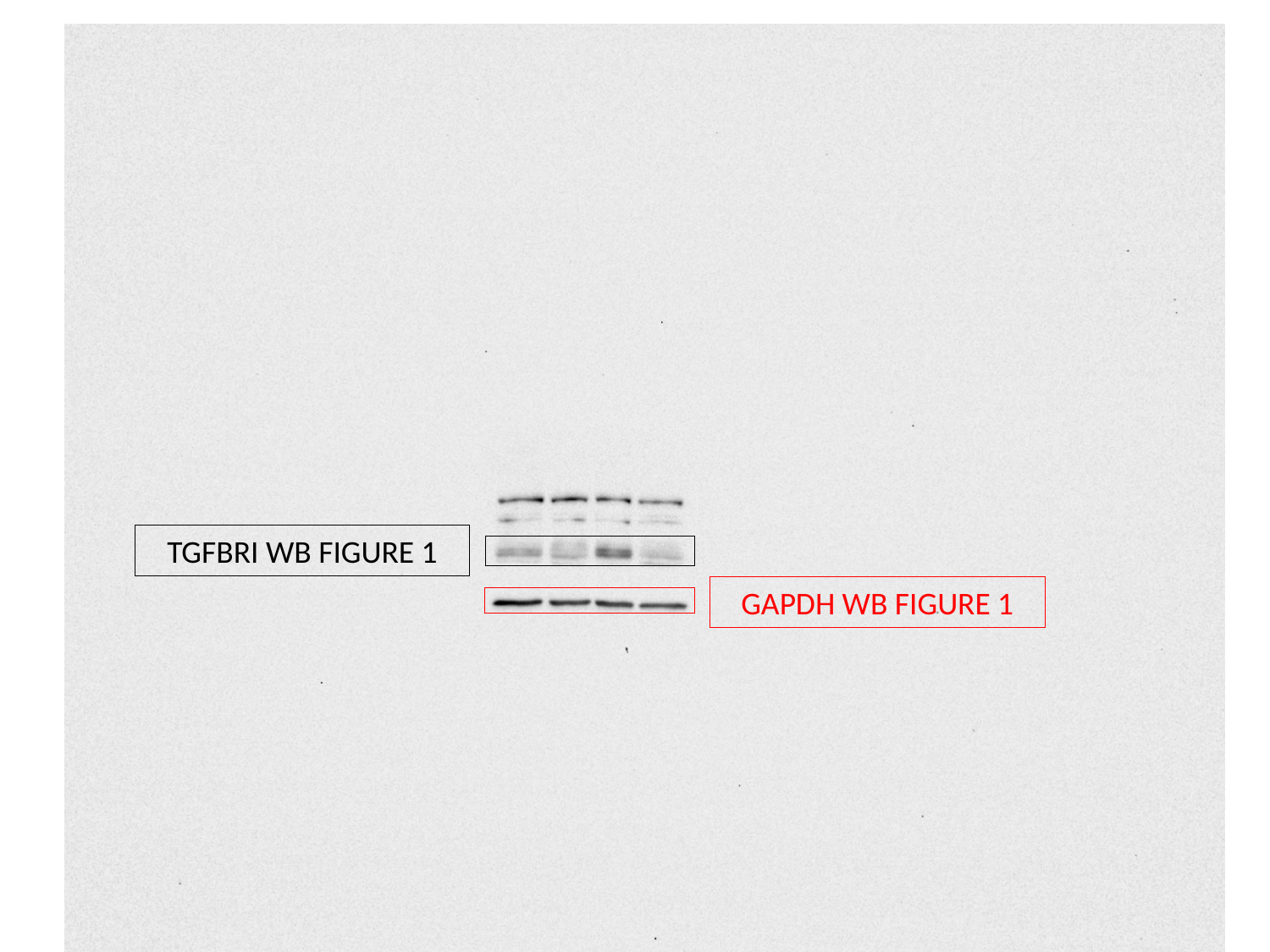

TGFBRI WB FIGURE 1
GAPDH WB FIGURE 1

## Slide 2
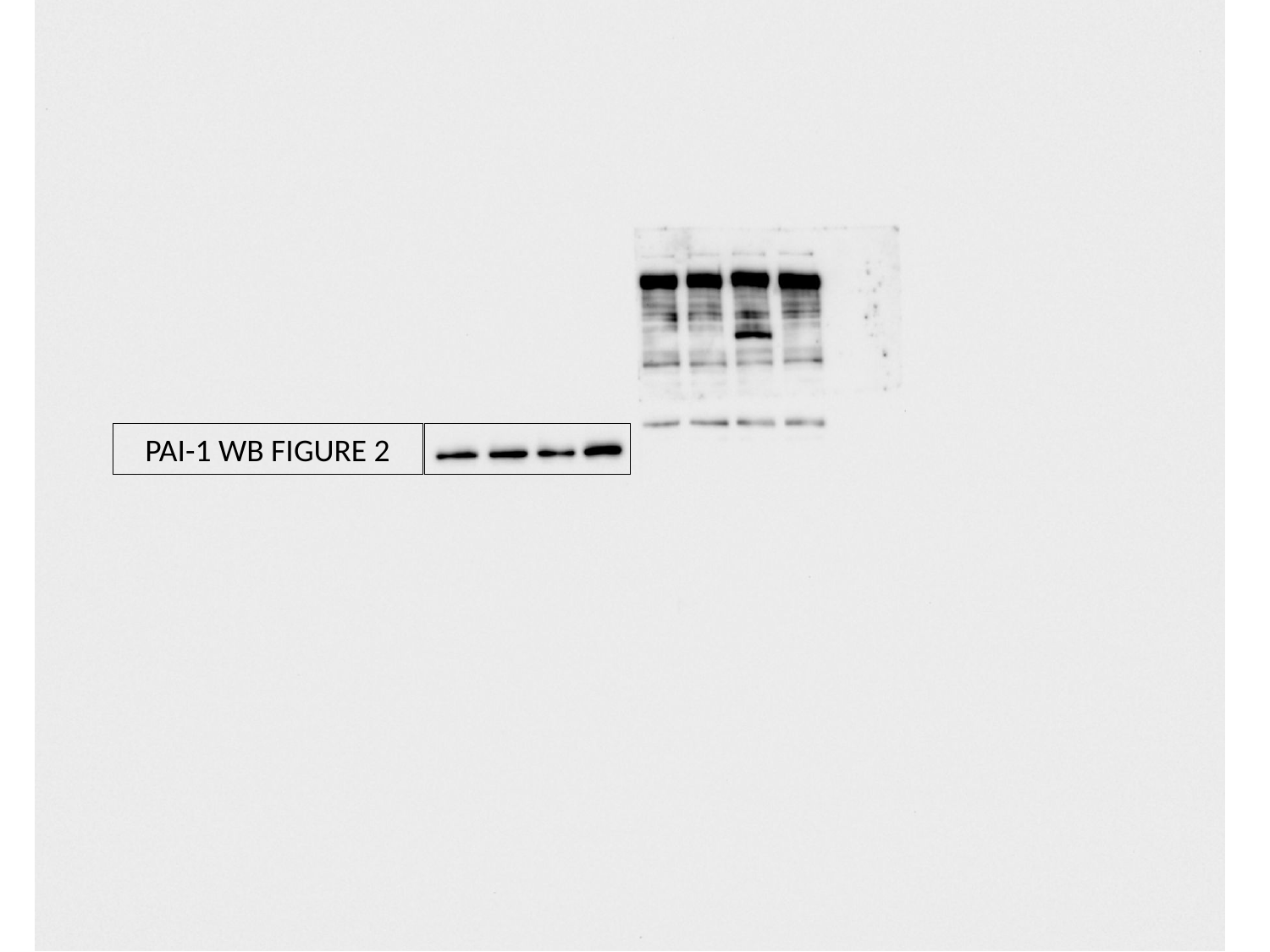

PAI-1 WB FIGURE 2

## Slide 3
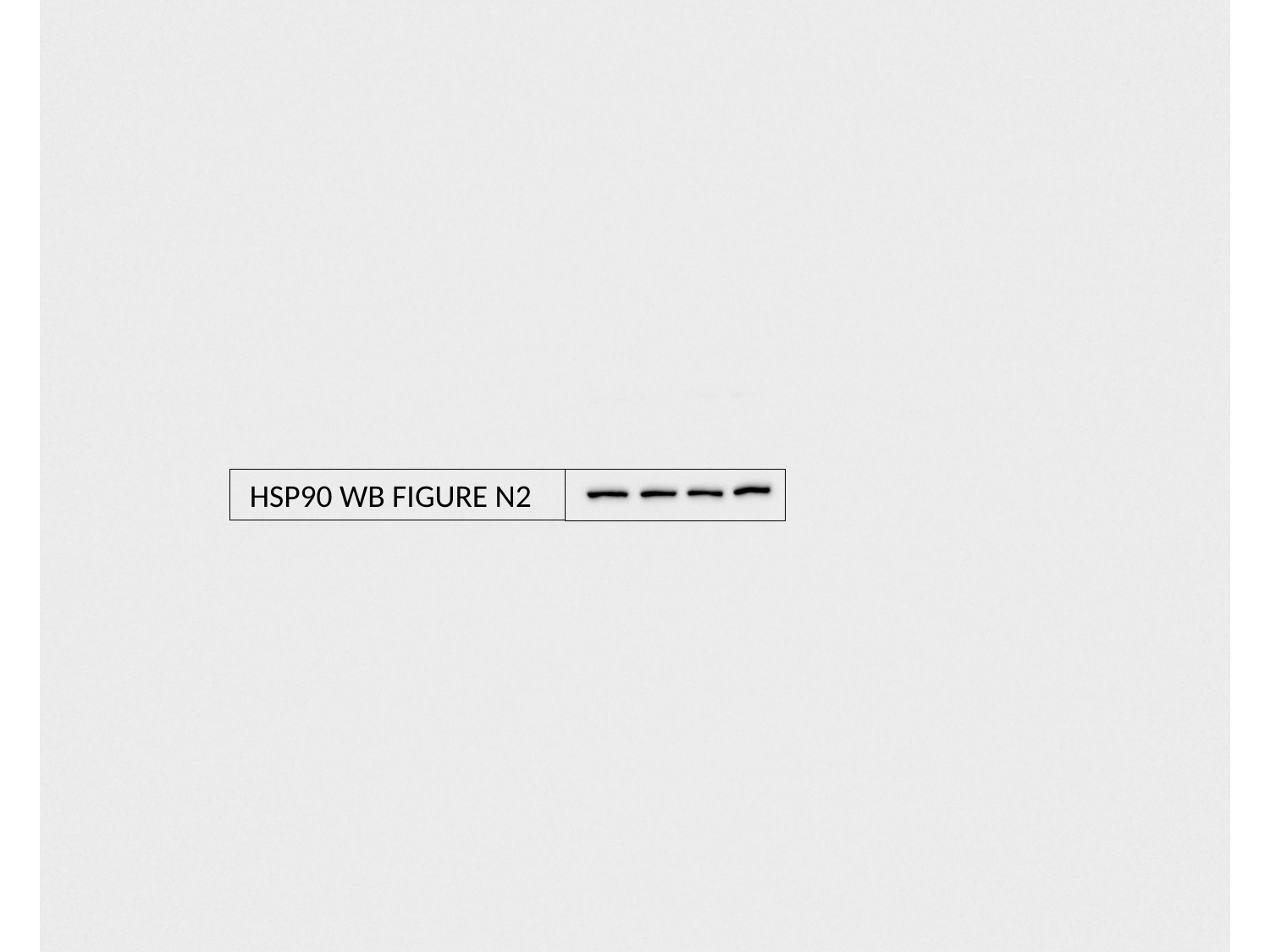

HSP90 WB FIGURE N2

## Slide 4
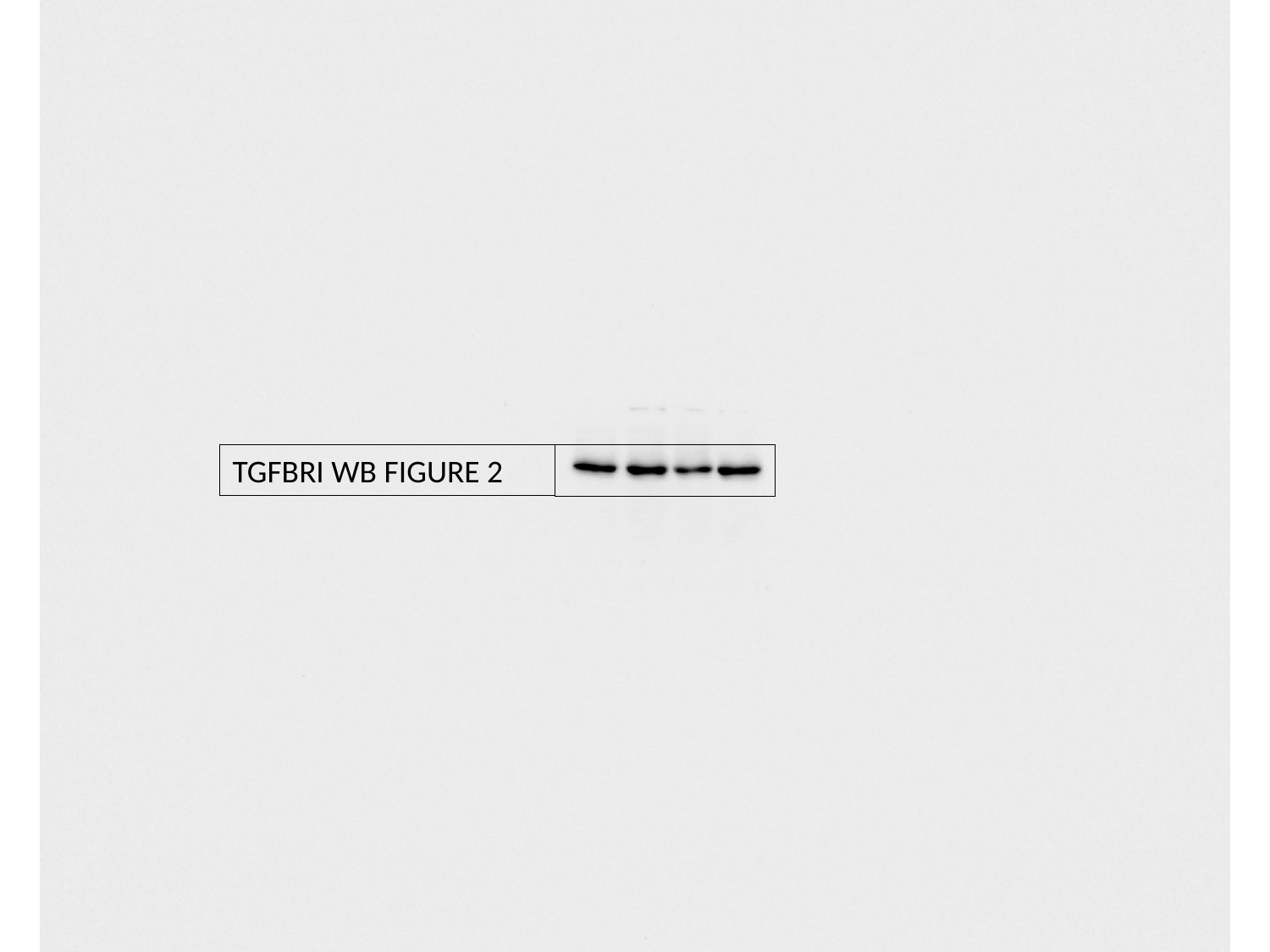

TGFBRI WB FIGURE 2

## Slide 5
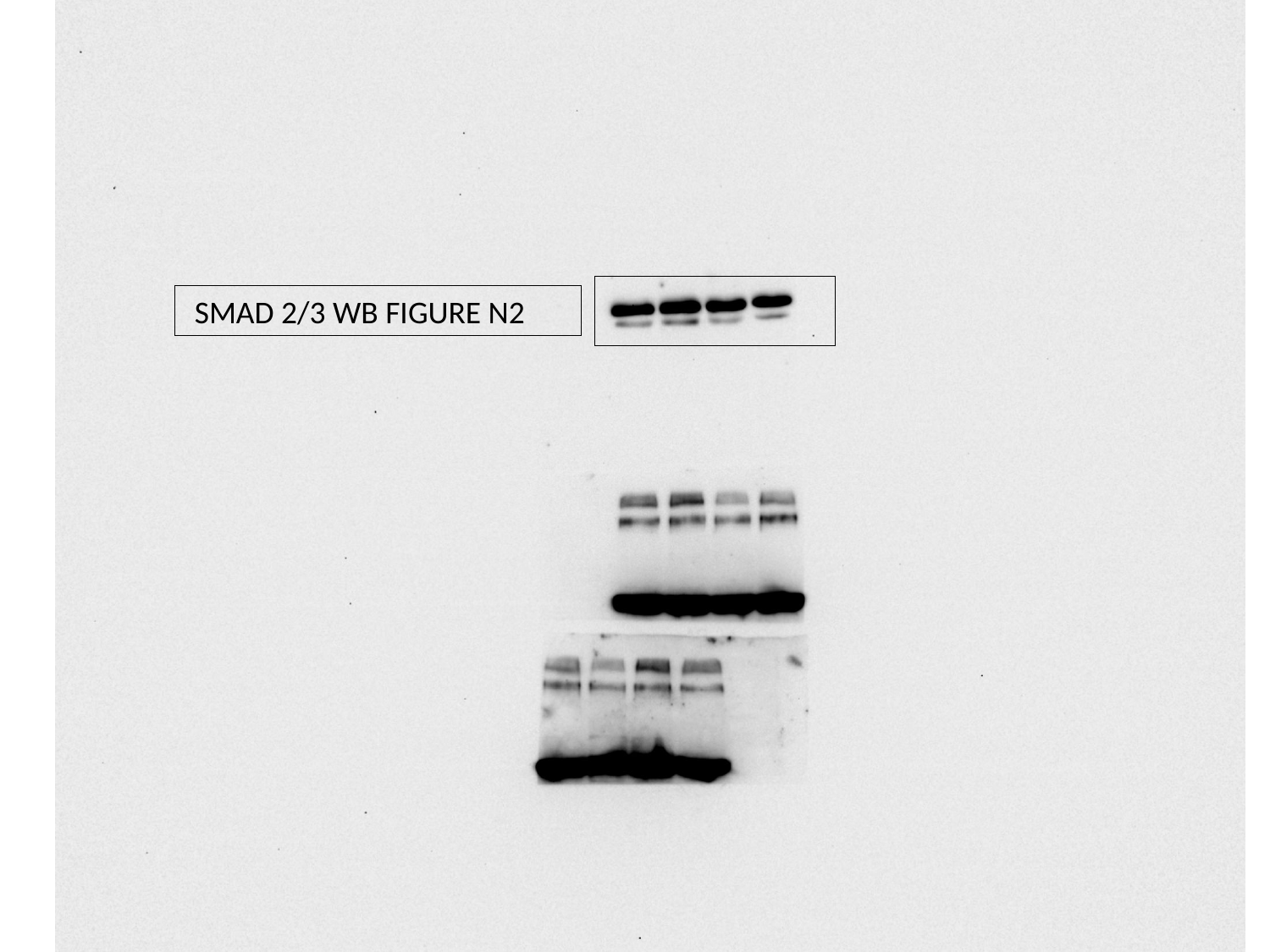

SMAD 2/3 WB FIGURE N2

## Slide 6
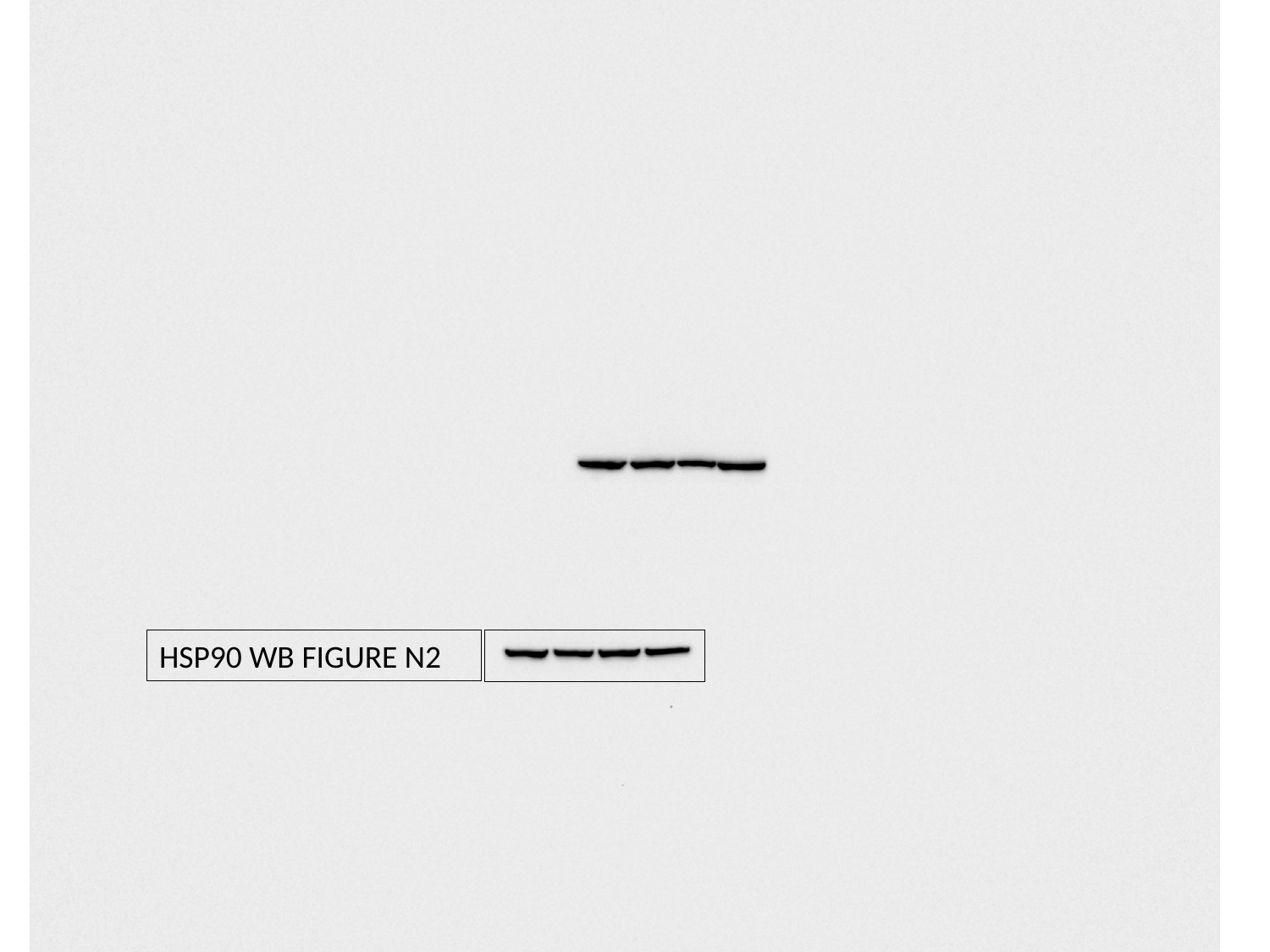

HSP90 WB FIGURE N2

## Slide 7
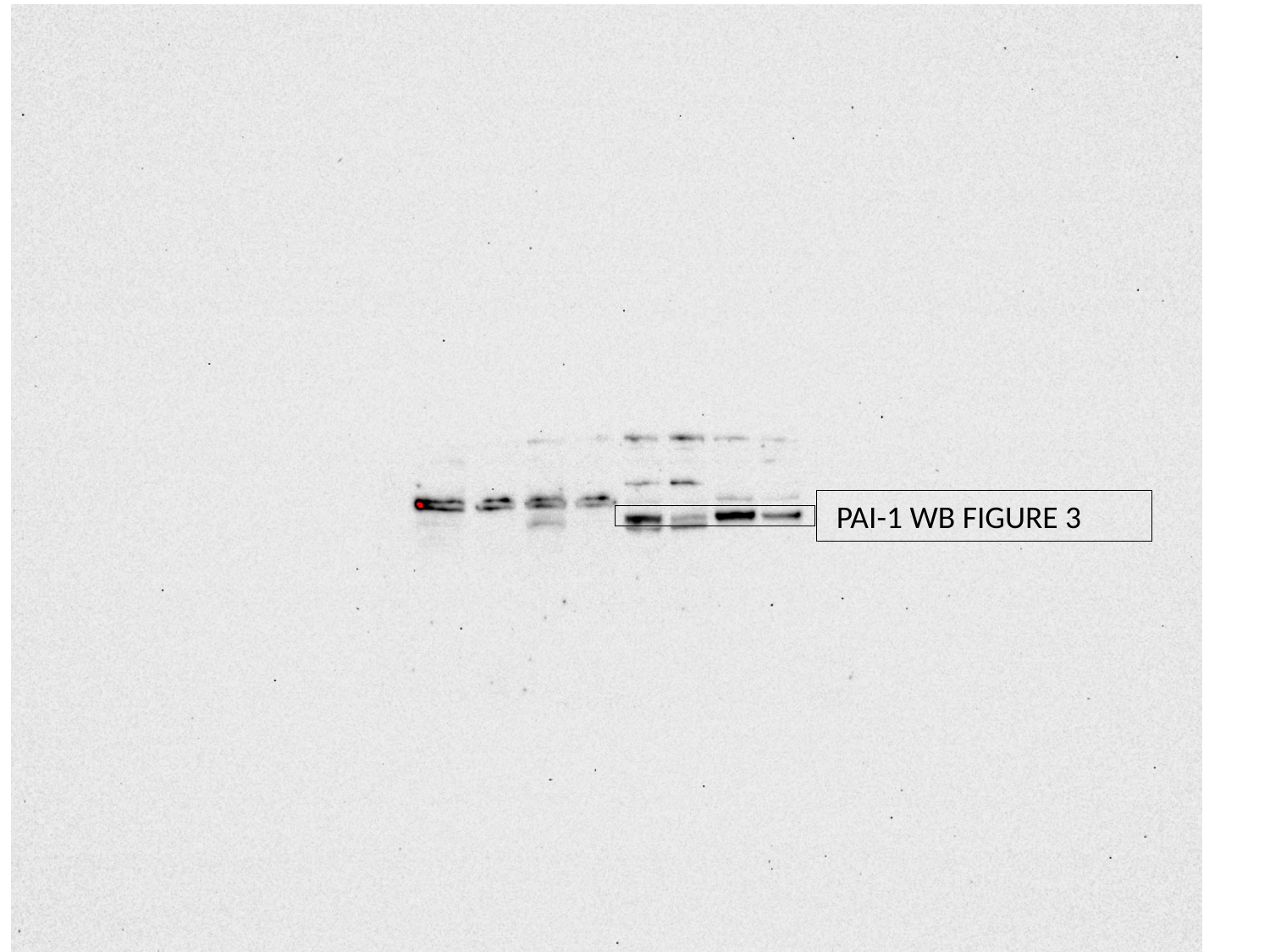

PAI-1 WB FIGURE 3

## Slide 8
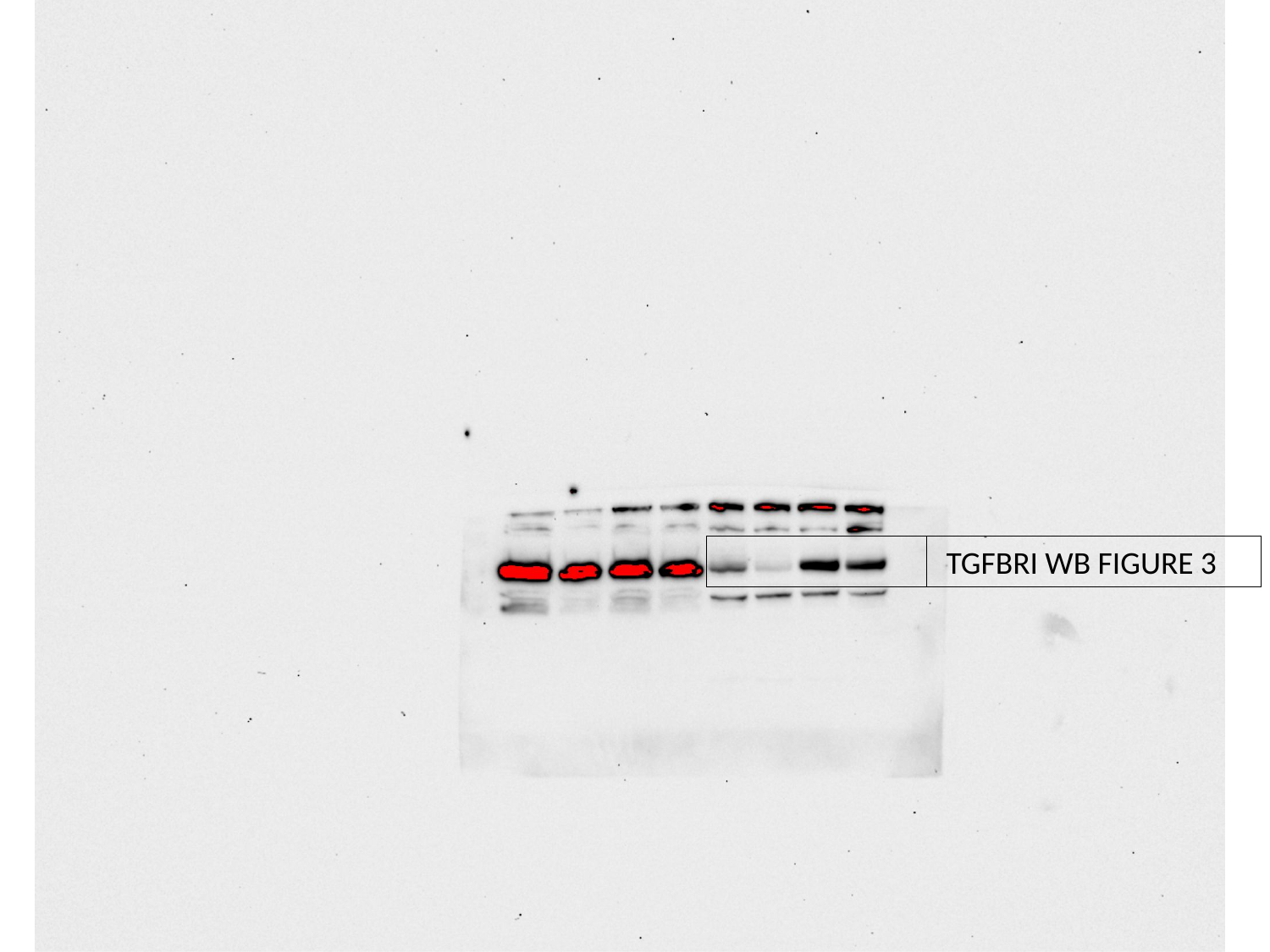

TGFBRI WB FIGURE 3

## Slide 9
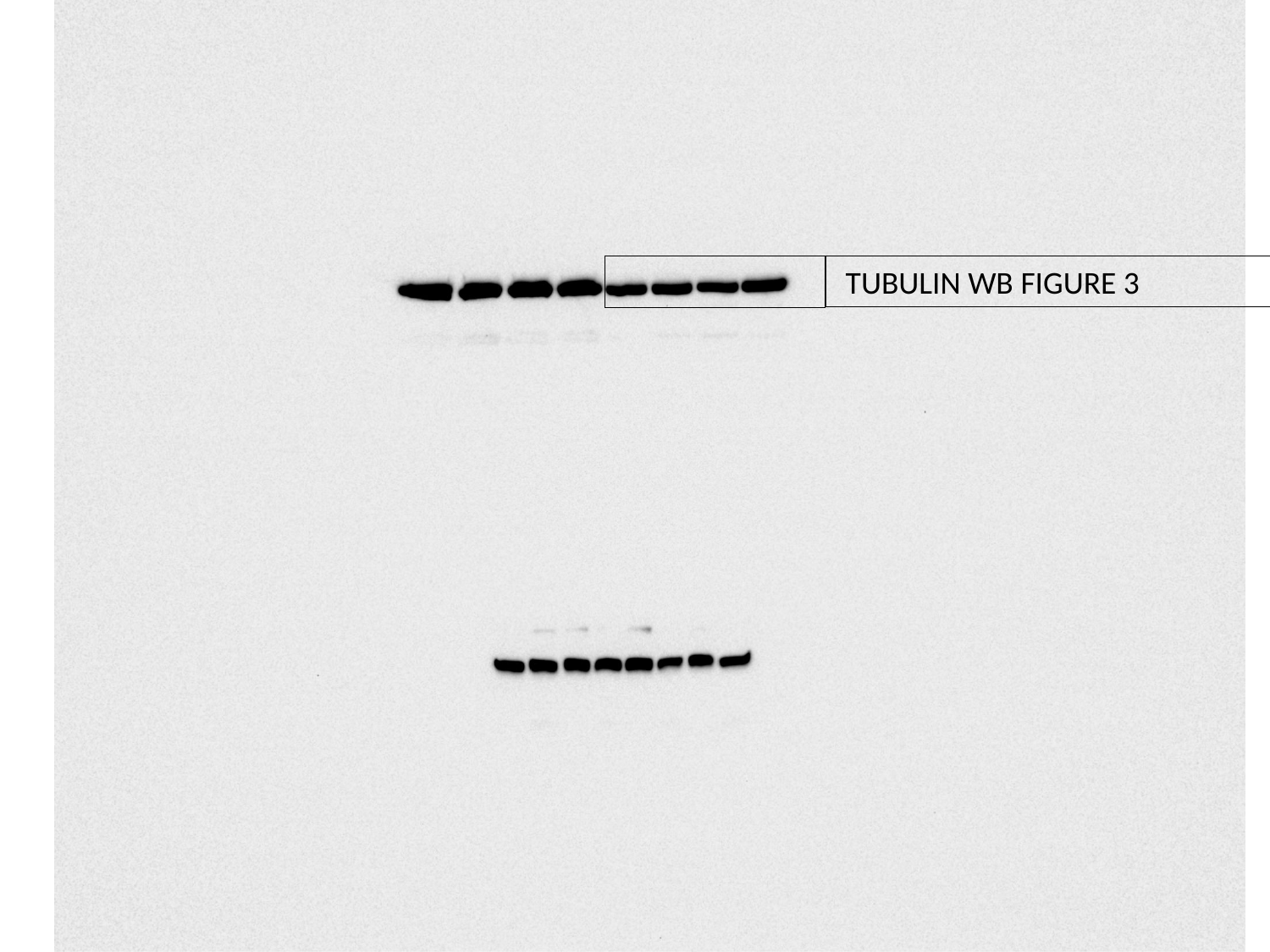

TUBULIN WB FIGURE 3

## Slide 10
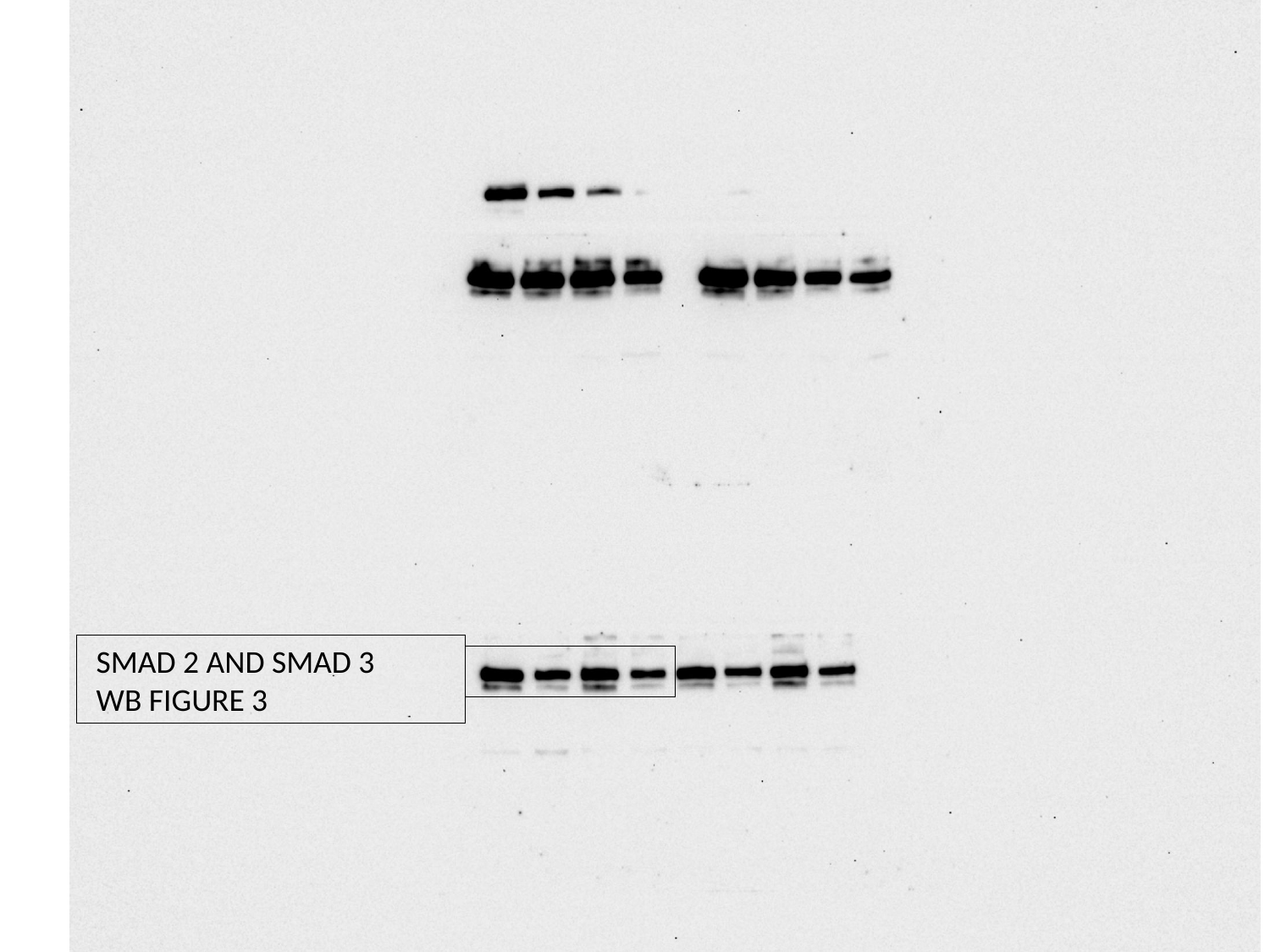

SMAD 2 AND SMAD 3
 WB FIGURE 3

## Slide 11
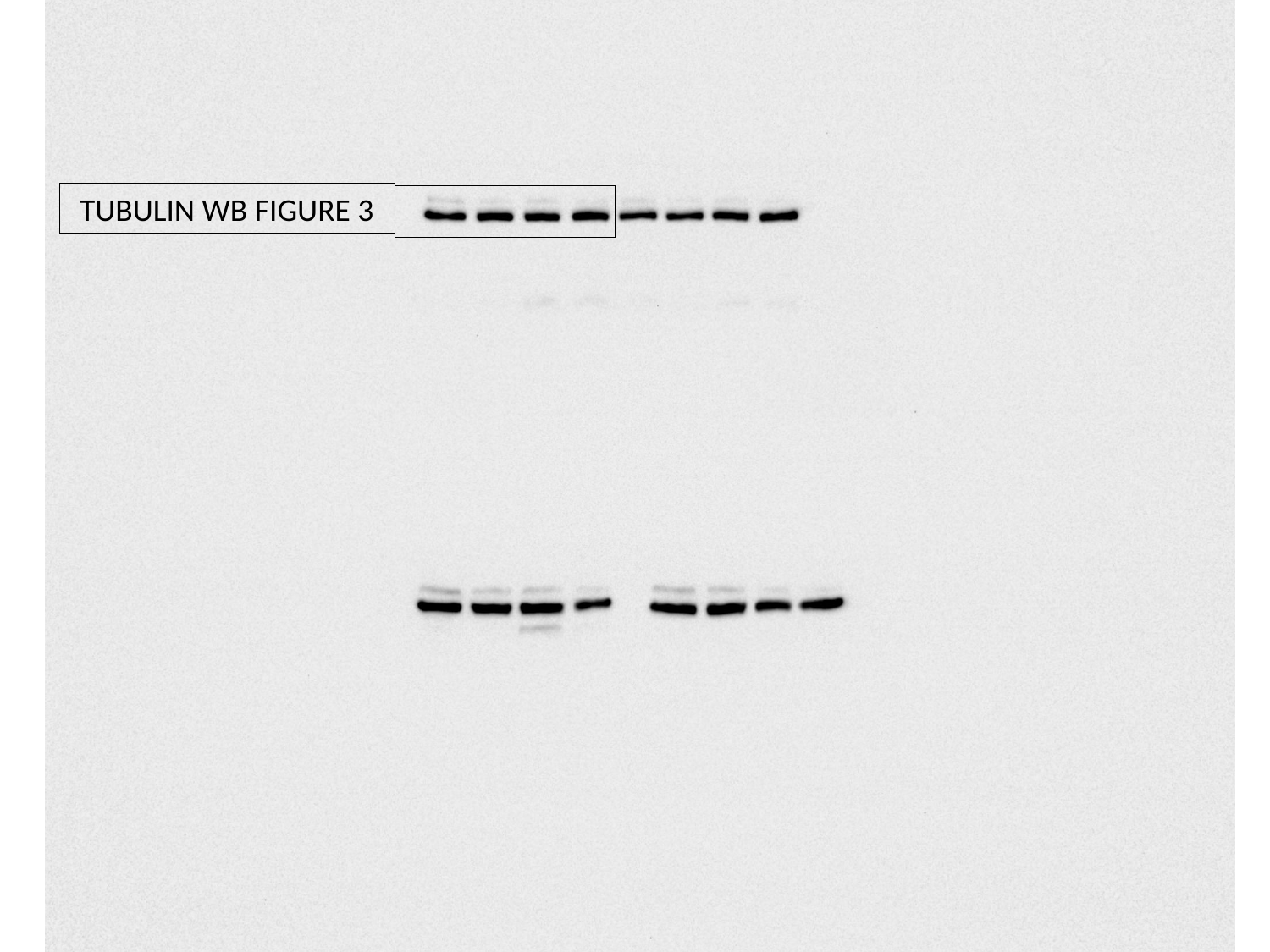

TUBULIN WB FIGURE 3

## Slide 12
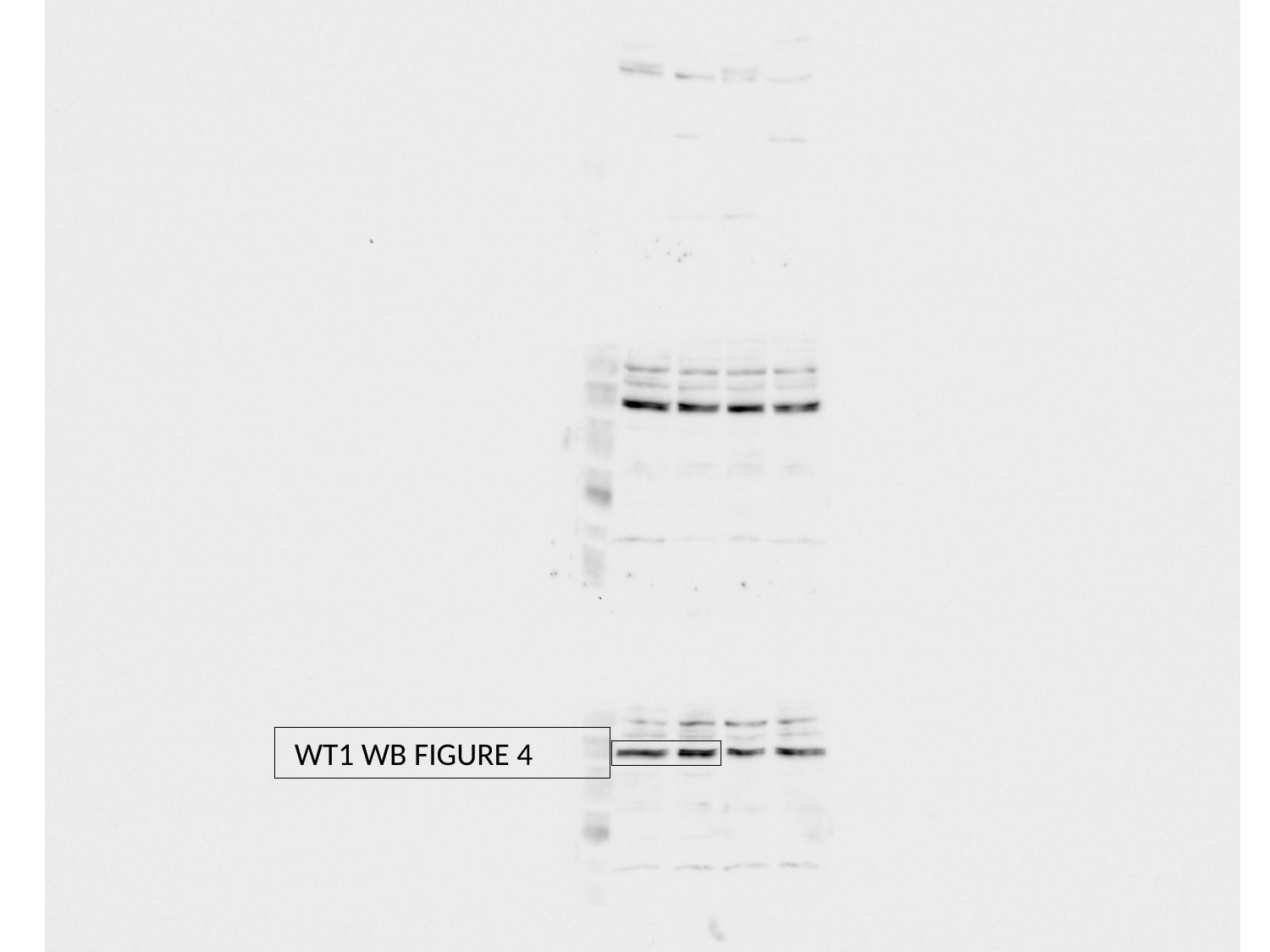

WT1 WB FIGURE 4

## Slide 13
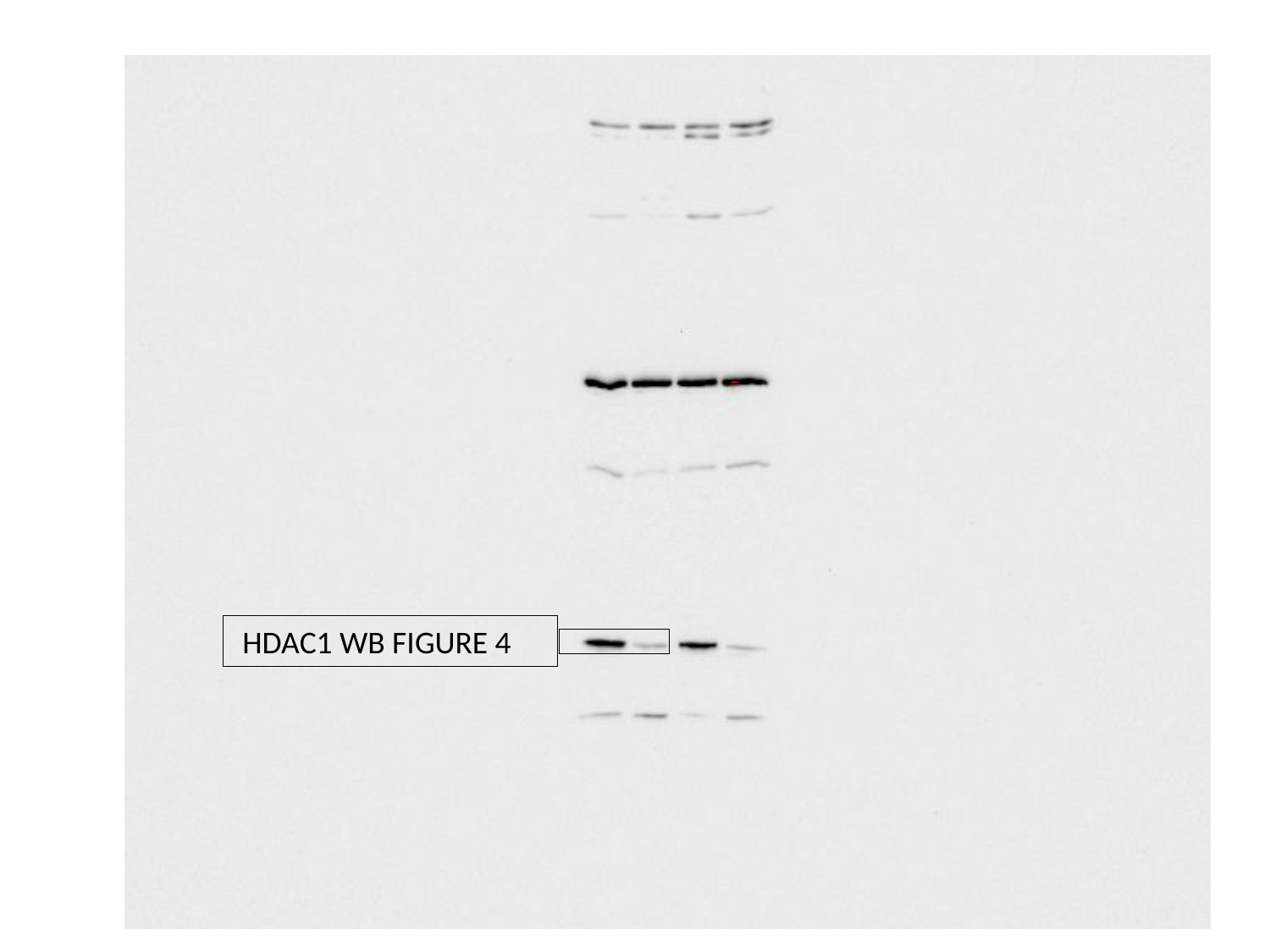

#
 HDAC1 WB FIGURE 4

## Slide 14
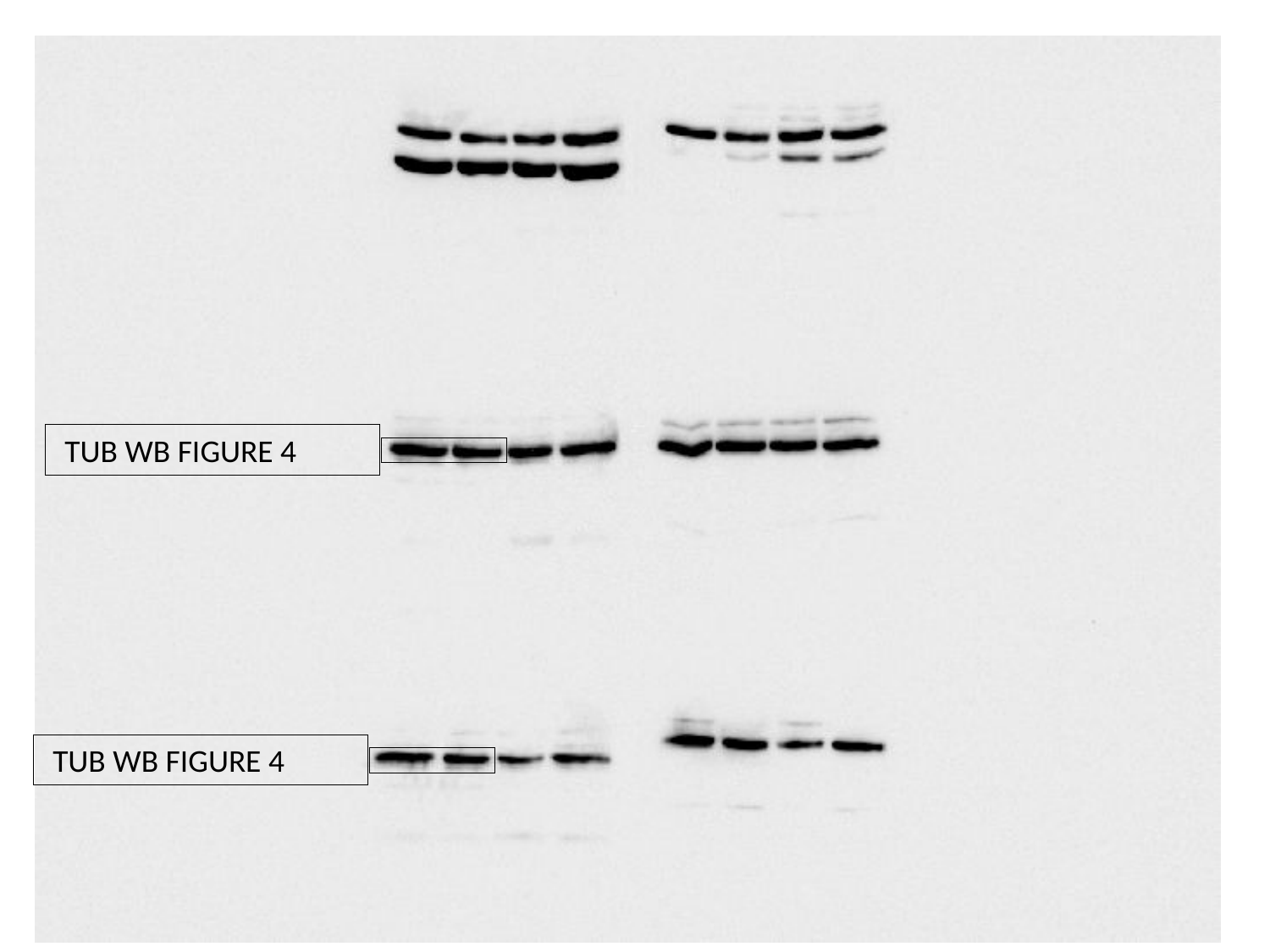

#
 TUB WB FIGURE 4
 TUB WB FIGURE 4

## Slide 15
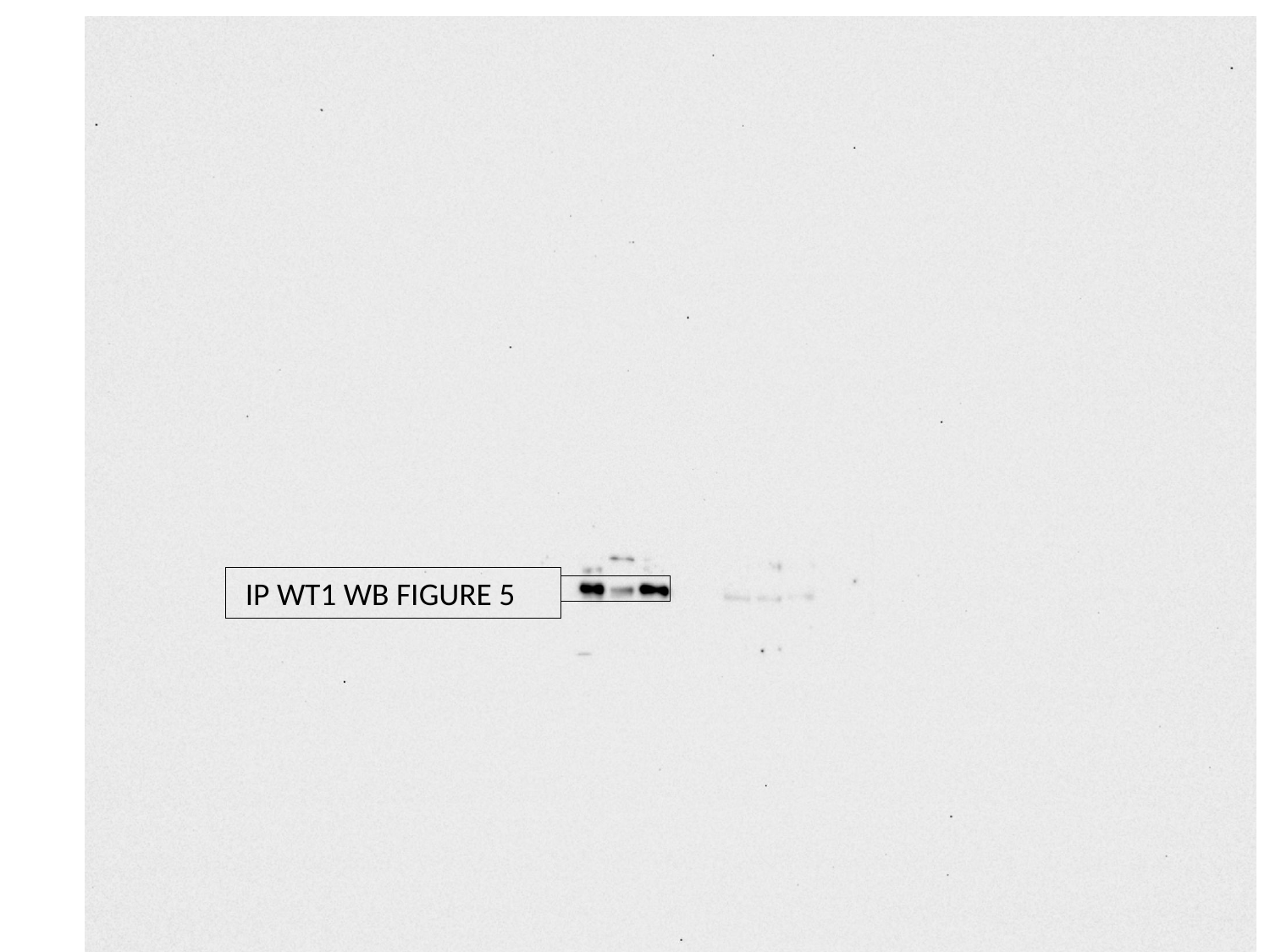

IP WT1 WB FIGURE 5

## Slide 16
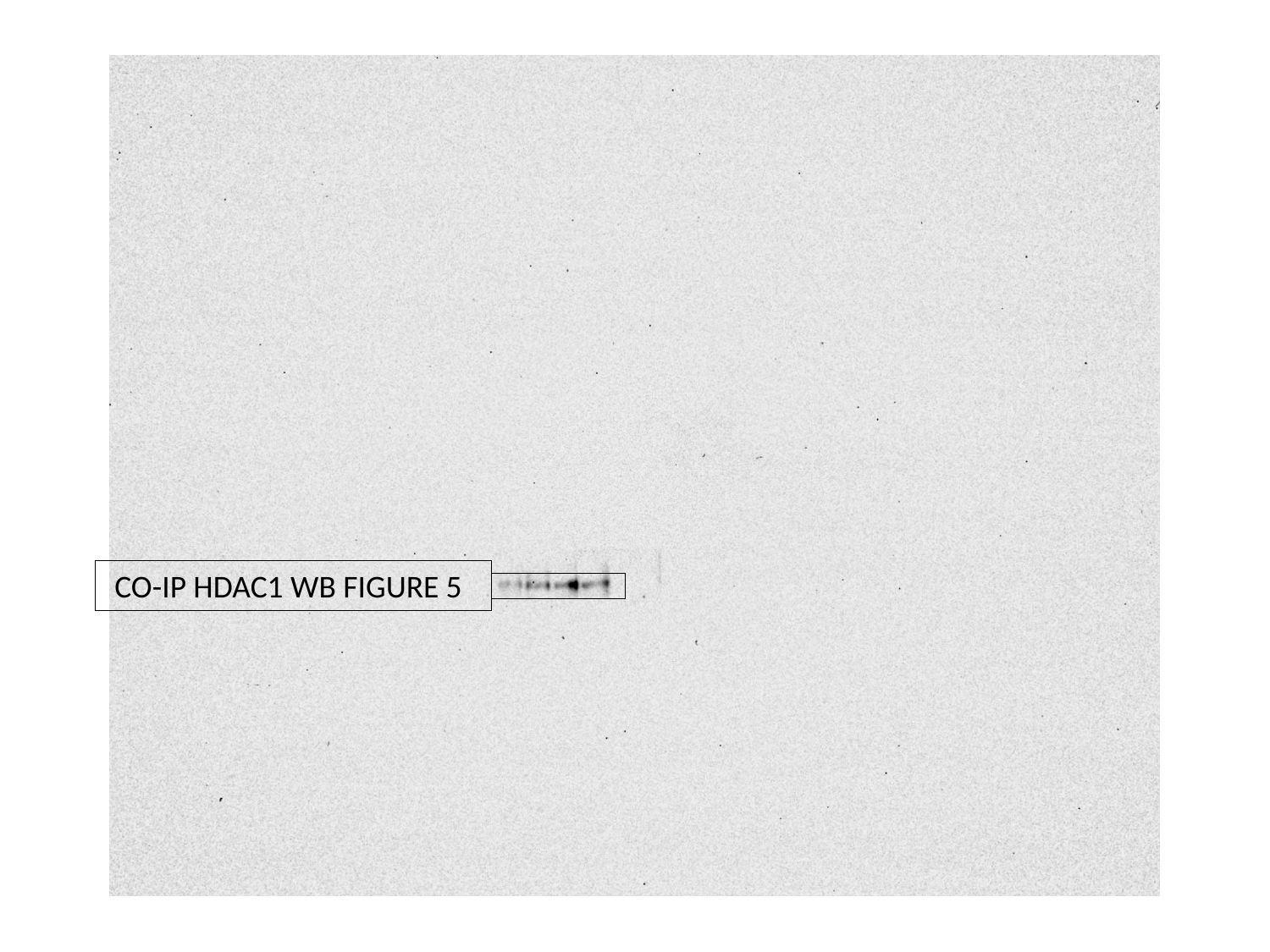

CO-IP HDAC1 WB FIGURE 5

## Slide 17
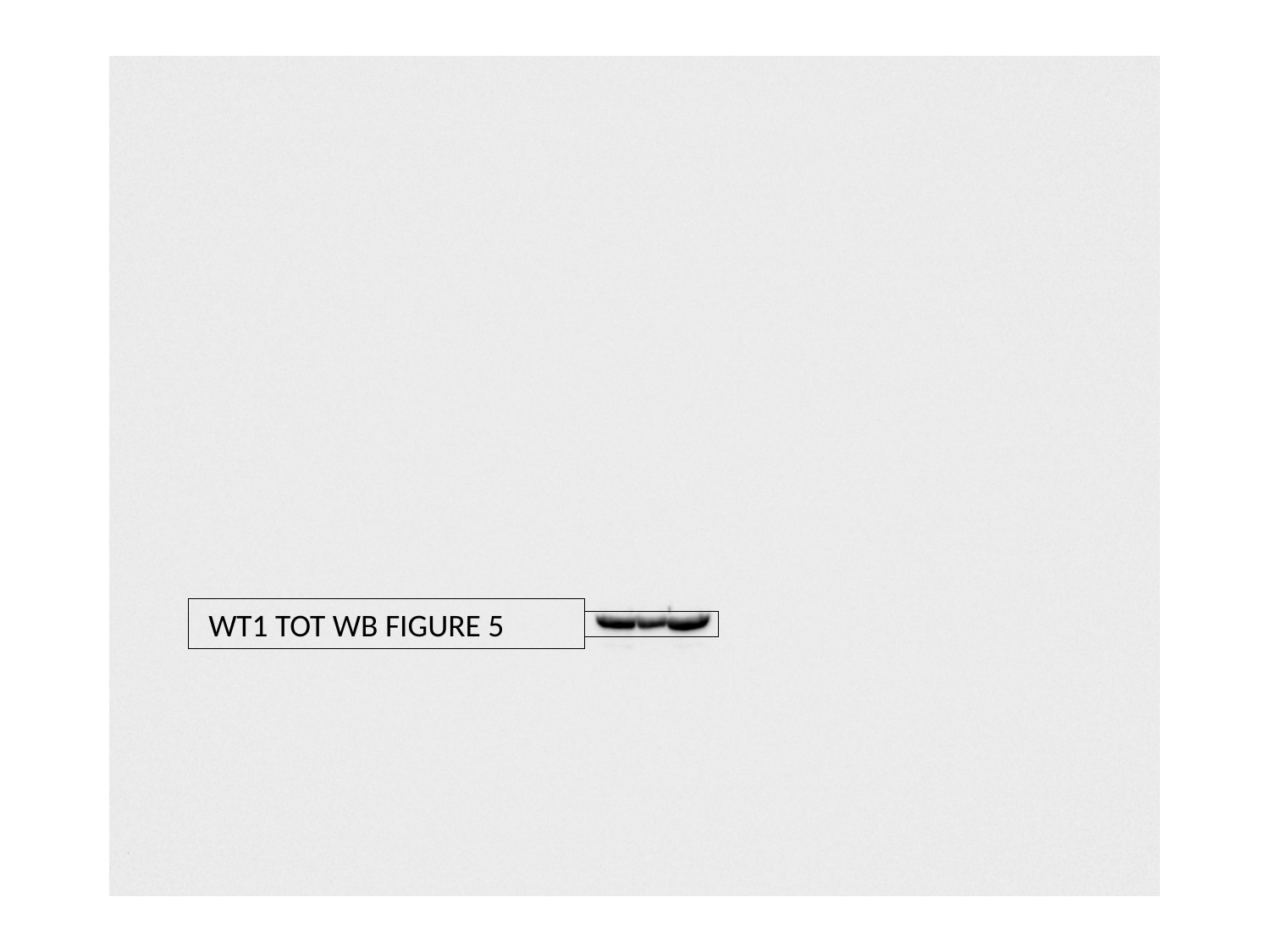

WT1 TOT WB FIGURE 5

## Slide 18
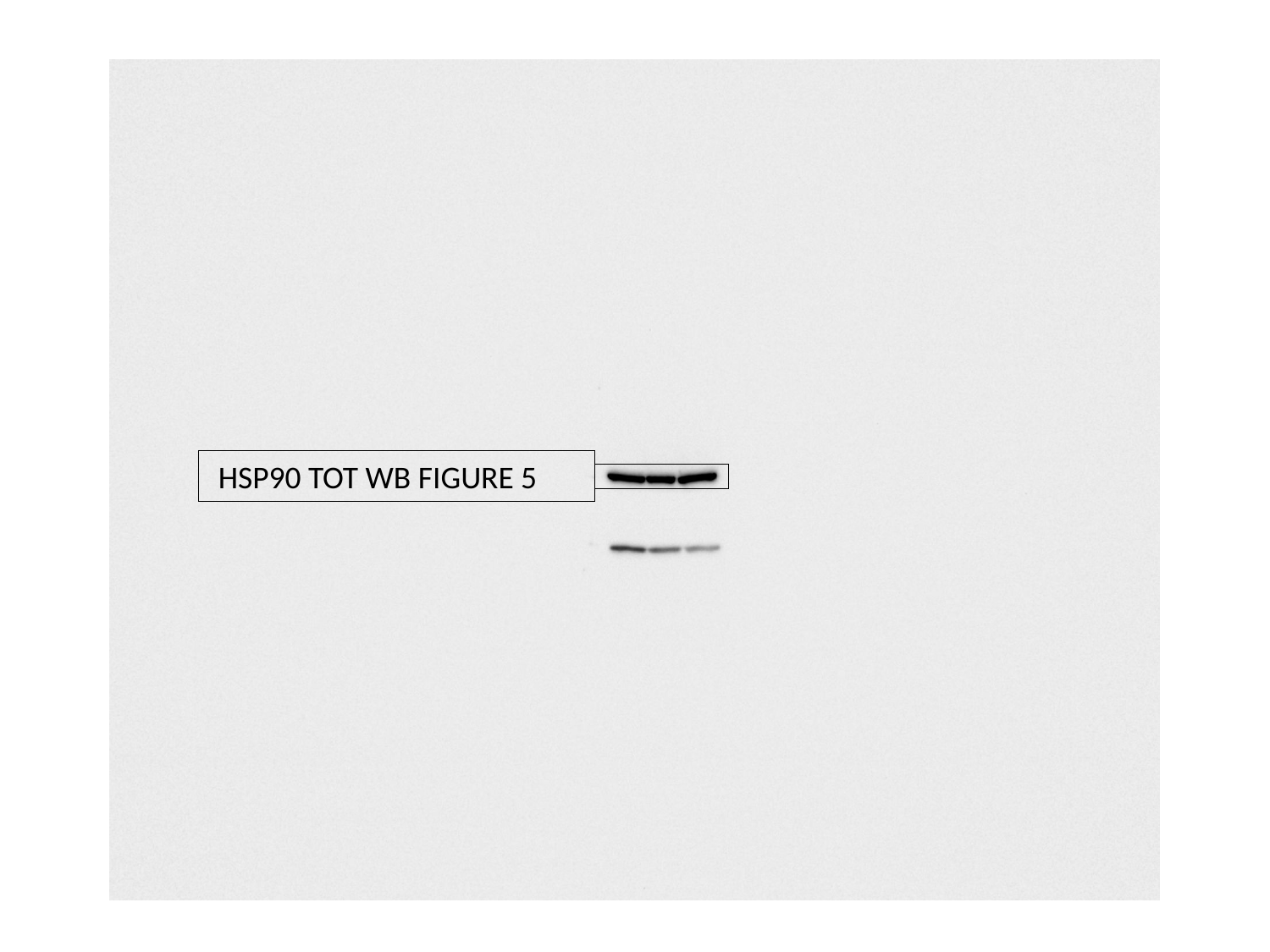

HSP90 TOT WB FIGURE 5

## Slide 19
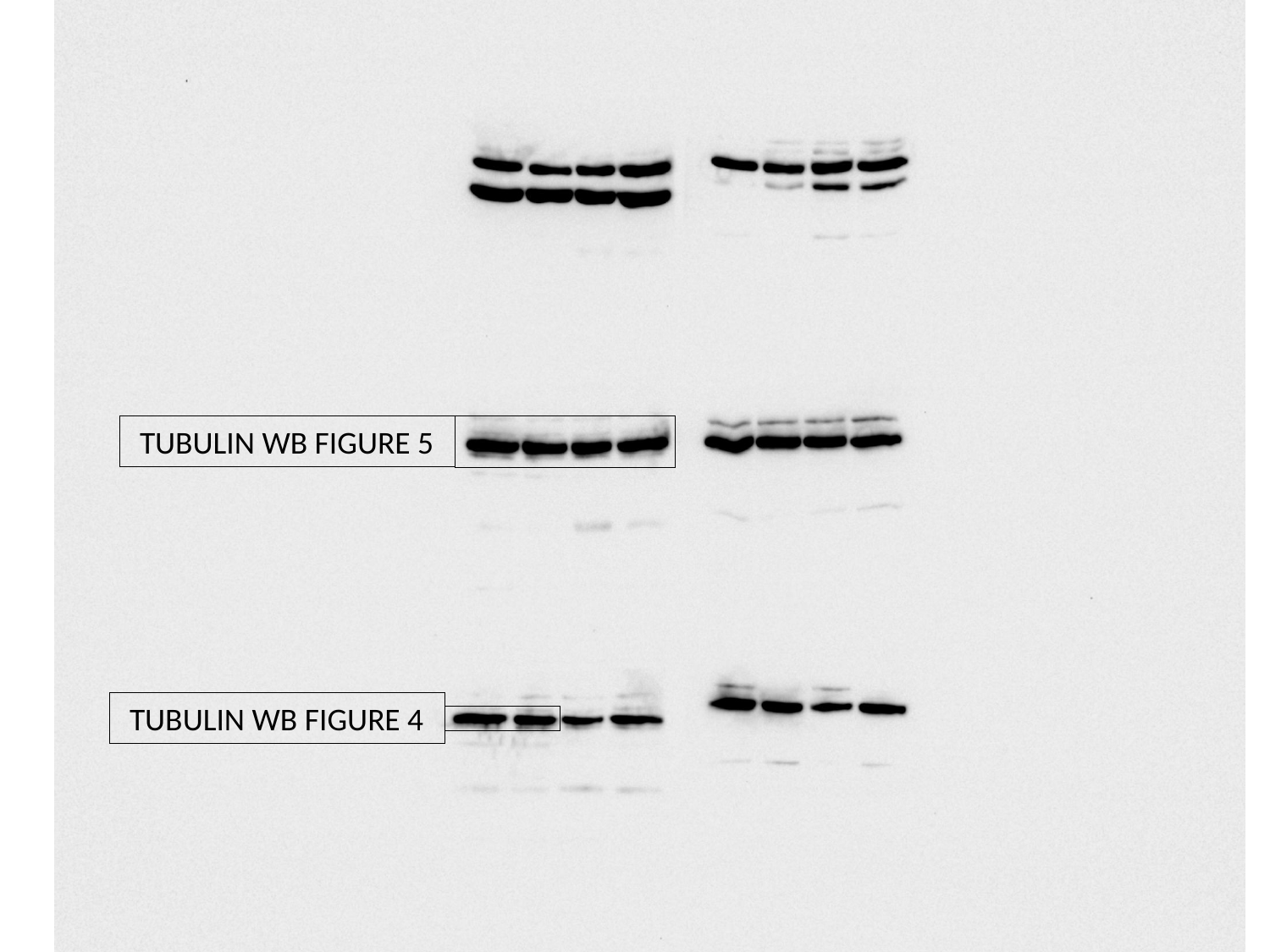

TUBULIN WB FIGURE 5
 TUBULIN WB FIGURE 4

## Slide 20
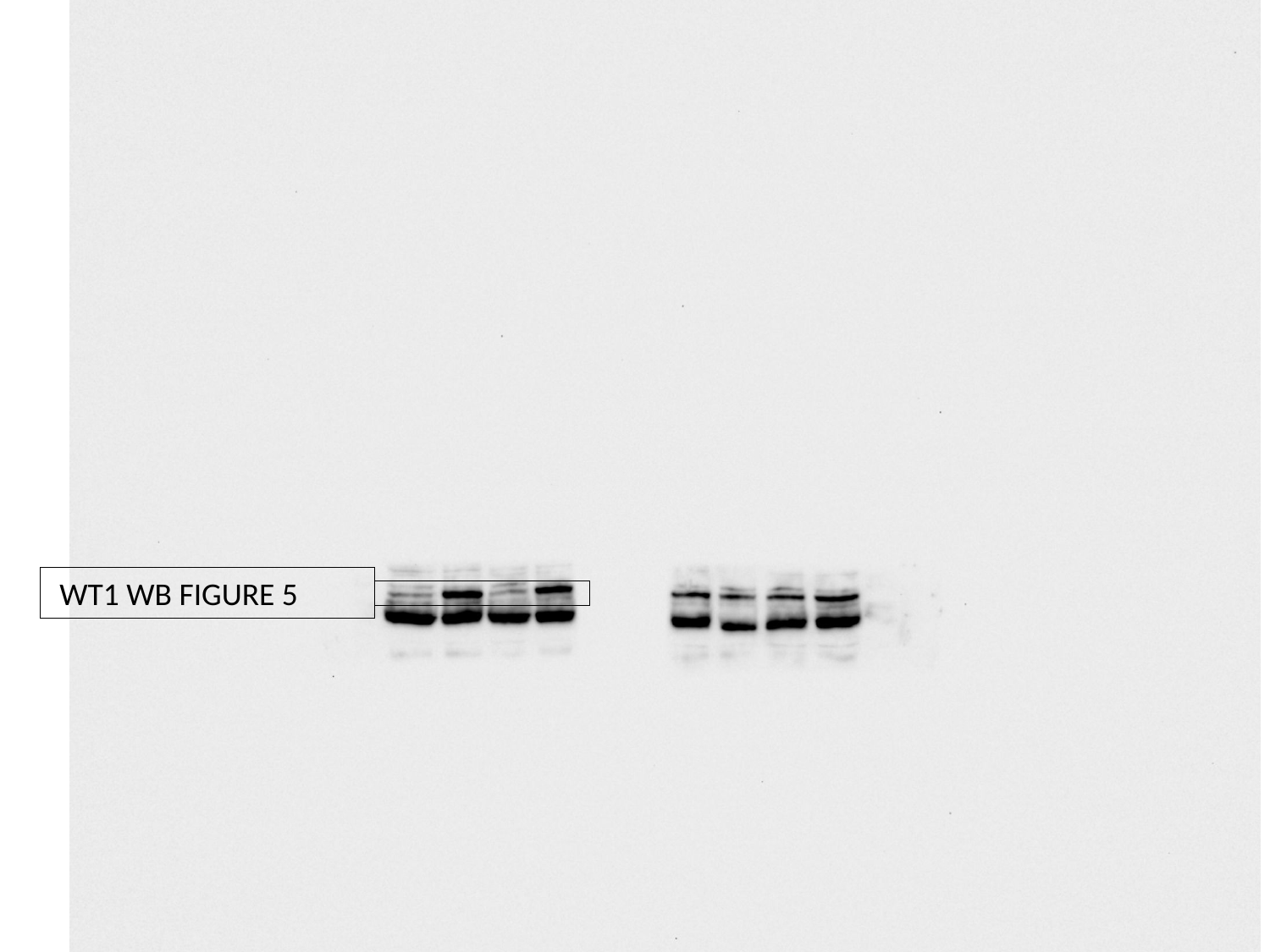

WT1 WB FIGURE 5

## Slide 21
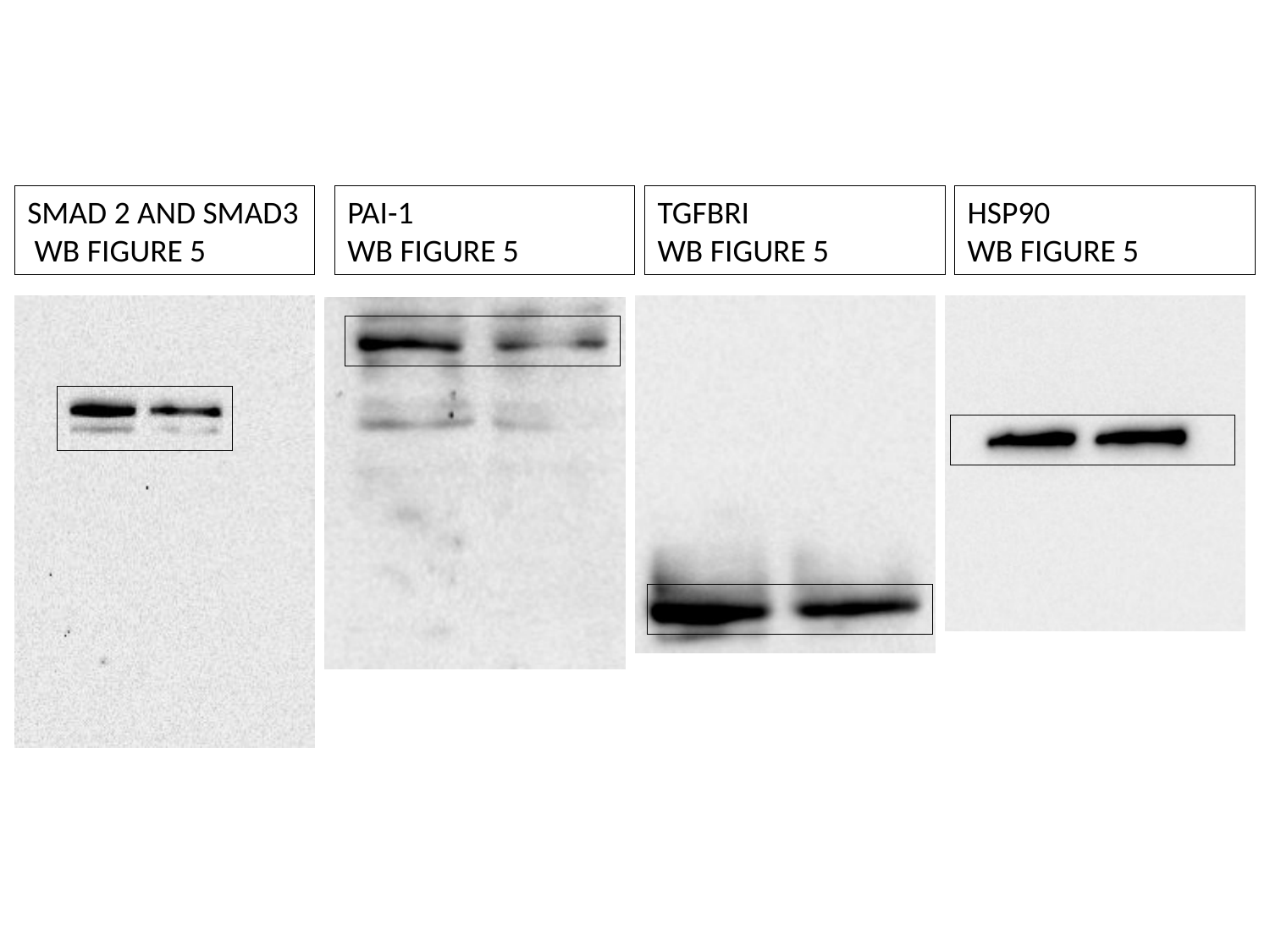

PAI-1
WB FIGURE 5
SMAD 2 AND SMAD3 WB FIGURE 5
TGFBRI
WB FIGURE 5
HSP90
WB FIGURE 5

## Slide 22
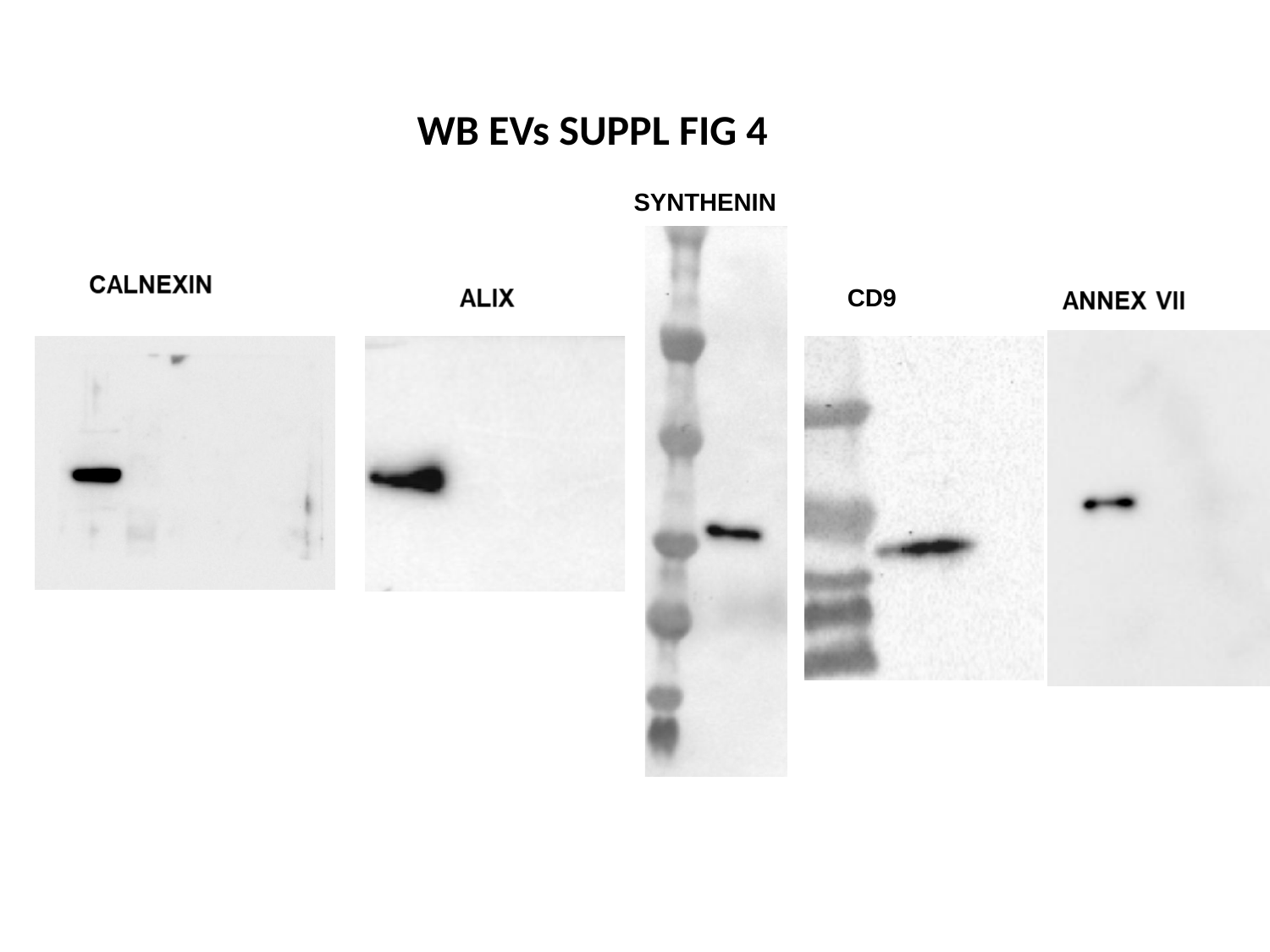

WB EVs SUPPL FIG 4
SYNTHENIN
CD9
